# When the City Speaks: How Urban Form Shapes the Cultural Transmission of Spatial Conceptualization

**DOI:** 10.1162/OPMI.a.380

**Published:** 2026-08-13

**Authors:** Zahra Alinam, Riccardo Fusaroli, Jonas M. Nölle, Cordula Vesper, Kristian Tylén

**Affiliations:** Department of Coevolution of Land Use and Urbanisation, Max Planck Institute of Geoanthropology, Jena, Germany; Department of Linguistics, Cognitive Science and Semiotics, Aarhus University, Aarhus, Denmark; Interacting Minds Centre, Aarhus University, Aarhus, Denmark; Centre for Language Evolution Studies, Nicolaus Copernicus University in Toruń, Toruń, Poland; School of Psychology & Neuroscience, University of Glasgow, Glasgow, UK

**Keywords:** spatial cognition, cognitive-cultural niche, conceptualization, spatial language, urban affordances, wayfinding

## Abstract

How do urban environments shape the way people conceptualize and communicate space? Using a cultural transmission experiment in virtual reality, we asked participants to navigate one of two contrasting cityscapes—a regular, grid-like “Manhattan” or an irregular, landmark-rich “Barcelona”—and to produce route descriptions and sketch maps. We examined participants’ spatial conceptualizations and tracked how spatial communication was transmitted across six simulated generations. Results show that city layout shaped spatial language and map production. Participants used more horizontal (e.g., numbered routes and intersections) descriptors in the “Manhattan” environment and more vertical (e.g., buildings distinguished by colors and textures) descriptors in the “Barcelona” environment. These strategies were also more effective: participants completed navigation tasks faster when the descriptions they received matched the structure of the environment. We observed robust cultural transmission of spatial conventions, with participants’ productions predicted by the linguistic input they received. However, rather than showing cumulative elaboration over time, these conventions stabilized quickly. Individual-level analyses revealed consistency between language and maps, as well as across environments: participants formed coherent spatial models that persisted and shaped adaptation to a new cityscape. This suggests that individual cognitive stability may act as a mechanism for preserving, rather than progressively refining, cultural conventions. Together, these findings highlight urban environments as cognitive–cultural niches that shape how people perceive, conceptualize, and communicate space. We discuss implications for theories of spatial language, cultural evolution, and urban design, showing how city layout shapes not only navigation behavior, but also the structure of spatial thought.

## INTRODUCTION

In cities, daily practices of movement, coordination, and communication are deeply shaped by infrastructure, density, and the rhythms of collective life. Streets, transit systems, and public squares necessitate shared norms for how people conceptualize and navigate space (Jacobs, 1961; Simmel, 2002). Here, we define ‘conceptualization’ as how people understand spatial environments and how they describe, communicate, and represent that understanding (Walmsley, 1988)—for instance through language or maps. According to theories of grounded cognition, conceptualizations are not only shaped by innate cognitive mechanisms but emerge from the continuous interaction of sensory-motor systems, the physical environment, and the social world (Barsalou, 2015; Tylén et al., 2013). The notion of affordances captures this relationship; affordances are possibilities for action provided to an organism by the environment given the organism’s particular sensorimotor, perceptual and cognitive abilities (Goldstein, 1981; Greeno, 1994). In urban environments, the physical organization of streets, buildings, and public spaces systematically channels movement, interaction, and attention, shaping how space is perceived, described and navigated (Alinam & Celani, 2026; Gehl, 1987; Jacobs, 1961; Scott, 2020). As people navigate these environments, they form and update conceptualizations that reflect these structural properties (Alinam et al., 2023; Tylén et al., 2021). Previous work on urban legibility suggests that spatial layouts differ in how easily they can be organized and interpreted by users (Lynch, 1960), providing a theoretical basis for expecting differences in spatial strategies across environments.

This raises a central question: How do the material affordances of urban environments—such as grid layouts or landmark configurations—shape how individuals and communities conceptualize and communicate about space, both in the moment and through the emergence and transmission of cultural conventions?

To address this question, we propose that spatial language and the underlying conceptualizations of cityscapes emerge from the dynamic interplay of three key forces. First, the physical affordances of the built environment shape how people conceptualize and describe space (Gehl, 1987; Jacobs, 1961; von Uexküll, 1921). Second, cultural traditions—including the linguistic conventions transmitted across individuals—influence how these strategies are learned and reused. In experimental research, such processes are often studied using transmission chain (or iterated learning) paradigms, where information is passed from one participant to the next, allowing researchers to track how representations change or stabilize over successive ‘generations’. Critically, this paradigm provides a controlled framework for creating counterfactual scenarios, for example, whether transmission merely involves copying the previous generation, establishing a shared identity (“this is the way we do things here”), or producing effective instructions. Thus, transmission chain paradigms can enable researchers to examine how cognitive biases, communicative functions and other contextual variables shape the emergence and stabilization of shared representations (Kirby et al., 2008; Mesoudi & Whiten, 2008; Pagnotta et al., 2024, 2026). In this context, cultural transmission refers to the process by which information is passed between individuals, whereas cultural evolution concerns the population-level consequences of such processes over time, including the emergence and stabilization of shared patterns (Kirby et al., 2008; Mesoudi, 2011). While much formal work on cultural evolution has focused on transmission mechanisms, the selective processes that determine which variants are retained and propagated have received comparatively less attention (Smaldino, 2026). In the present study, the environmental manipulation and individual-level analyses allow us to examine the selection pressures that shape which spatial concepts are adopted, maintained, and carried over across contexts, while the transmission chain design isolates the pathway of transmission. Specifically, the linear, non-interactive structure of the present experiment focuses on transmission without real-time feedback (e.g., clarification or repair), which may emphasize direct transmission effects over opportunities for cumulative cultural change (Fay et al., 2018; Pagnotta et al., 2024). Third, individual cognition forms the basis for transmission: in transmission chain paradigms, each participant’s output becomes the input for the next, allowing individual-level biases to propagate and shape shared representations over successive generations. In this process, individuals act as “bottlenecks,” meaning that information is filtered through limited cognitive capacities (e.g., memory and attention), resulting in a partial compression of the information they receive, so that not all aspects of the input are preserved as it is passed on (Bartlett, 1932; Griffiths et al., 2008; Kalish et al., 2007; Tamariz & Kirby, 2015).

Amongst the most powerful vehicles for shared conceptualizations is language. Linguistic conventions emerge and stabilize through repeated use and mutual expectations within social groups, helping communities coordinate and communicate about their environments (Clark, 1996; Fusaroli et al., 2012; Hawkins et al., 2019; Lewis, 1969). As a result, linguistic systems vary systematically across communities, reflecting differences in how people perceive and interact with their environments. Such variation has been documented across a range of domains, including phonology, semantics, and spatial language (Haspelmath, 2010; Kirby et al., 2015). This variation reflects adaptive responses both to internal pressures (e.g., expressivity, learnability, processing efficiency; Christiansen & Chater, 2008; Kirby, 2017; Zipf, 1949) and to external pressures from the physical and social environment (Dale et al., 2013; Fusaroli et al., 2014a, 2014b; Lupyan & Dale, 2016; Tylén et al., 2013).

In this sense, language can be understood as a complex adaptive system, emerging from the interaction of multiple factors across individual and social levels, including cognitive constraints, communicative needs, and environmental pressures (Beckner et al., 2009). One influential account of this relationship is the linguistic niche hypothesis, which proposes that languages adapt to the socio-cultural and ecological environments in which they are used (Lupyan & Dale, 2010, 2016). Much of the evidence for this hypothesis comes from cross-cultural correlations between environmental factors and linguistic features, such as phoneme inventories in relation to climate (Everett, 2013, 2017; Maddieson & Coupé, 2015), basic color terms shaped by communicative needs and efficiency principles (Baronchelli et al., 2015; Brown & Lindsey, 2004; Gibson et al., 2017; Regier et al., 2016), communicative strategies shaped by the acoustic properties of the language (Dideriksen et al., 2023a, 2023b; Trujillo et al., 2023), event structure (Christensen et al., 2016; Majid et al., 2008; Malt et al., 2008), and spatial reference frames adapting to urban environments (Schlossberg, 2018). However, because these findings are largely correlational, they do not establish how such adaptations emerge or are maintained through cultural transmission, and may be confounded by alternative explanations such as shared language genealogy or areal contact (Roberts, 2018).

Spatial language offers a clear case for how language is shaped by environmental and sociocultural factors (Bohnemeyer et al., 2015; Nölle & Spranger, 2022; Palmer et al., 2017; Tylén et al., 2022). Communities employ different spatial frames of reference—such as egocentric (viewer-centered) or geocentric (environment-centered) systems—reflecting adaptation to their environments (Levinson, 2003; Majid et al., 2004; Pederson et al., 1998). Such variation demonstrates how environmental structure can shape the spatial concepts and linguistic strategies used for navigation and communication.

However, the degree to which community-level patterns are sustained depends not only on transmission but also on the consistency and flexibility of individual cognition. On the one hand, individual conceptualizations of space often show a high degree of intra-consistency—that is, stability across different tasks and contexts. For instance, proficiency in one configurational task, such as estimating directions, reliably predicts proficiency in other metric tasks, such as estimating straight-line distances or drawing accurate sketch maps, even in complex environments (Ishikawa & Montello, 2006). Individuals also form stable conceptual representations that are expressed across modalities like language and drawing (Barsalou, 2015). This suggests that individuals do not simply generate ad-hoc descriptions from environmental cues but maintain relatively enduring conceptualizations of space. On the other hand, cognition is not static. Cognitive functioning has been shown to vary across individuals and fluctuate within individuals over time as a consequence of learning and adapting to new environments, with within-person variability sometimes even exceeding differences between people (Gamaldo & Allaire, 2016; Vaughan & Birney, 2023). This combination of stability and flexibility makes individuals both carriers and transformers of spatial conceptualizations, constraining how conventions are learned, maintained, and adapted across contexts. Figure 1 illustrates this integrated framework.

**Figure 1.**
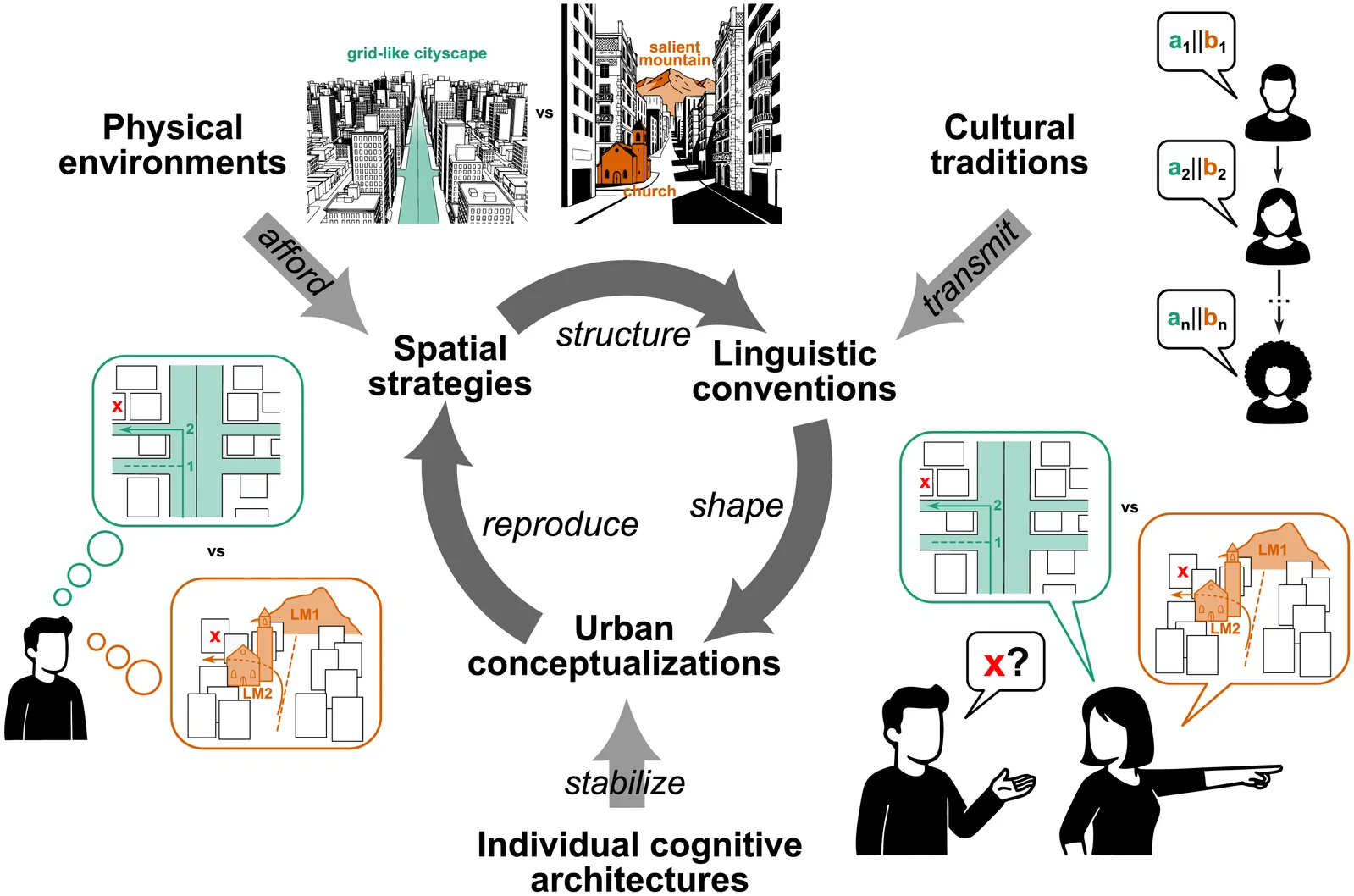
Dynamic feedback loop between physical environments, linguistic conventions, and individual cognitive architectures. The figure illustrates how distinct urban topographies—here a grid-like cityscape versus a city with salient vertical landmarks such as a mountain (LM1) and church (LM2)—support different spatial strategies. In a landmark-rich environment, a route description might say *“walk towards the mountain, then turn left after the church”*, whereas a grid-like layout supports horizontal, metric strategies such as *“follow the main road, then take the second street to the left”*. Such strategies shape the linguistic conventions used to describe space, which are socially transmitted across individuals as cultural traditions and become part of a community’s shared repertoire (strategies a or b on the top right). Through repeated use, these conventions become stabilized in individual cognitive representations of urban space, which in turn reinforce environment-specific conceptualizations and feed back into how new spatial strategies are formed. Note that this figure is a schematic illustration of the general principle; the actual experimental environments used buildings varying in color and texture rather than natural landmarks (see Figure 2).

Establishing causal links between environmental variables and spatial language remains challenging because of the complex interplay of influences and the limitations of observational data (Bohnemeyer et al., 2015; Roberts, 2018). Virtual reality (VR) offers a particularly powerful avenue for addressing this, combining high ecological validity with experimental control and immersive large-scale environments (Nölle & Peeters, 2025; Peeters, 2019). For example, prior work using VR and experimental tasks has shown that environmental affordances can shape the emergence of communicative conventions in spatial navigation (Nölle et al., 2020). Specifically, Nölle et al. (2020) demonstrated that different environmental structures systematically bias the linguistic strategies participants develop to describe space, with distinct environments promoting different conceptualizations (e.g., figural, line-based, or path-based descriptions). These conventions emerge through interaction and become increasingly stabilized over repeated trials.

In the present study, we extend this approach by incorporating a transmission chain paradigm, allowing us to examine not only how environment-dependent strategies emerge, but also how they are transmitted and maintained across individuals. In addition, we combine linguistic descriptions with sketch maps and focus specifically on urban environments rather than abstract mazes, enabling a more comprehensive and ecologically grounded analysis of spatial conceptualization across modalities, compared to earlier approaches that relied on photographs or tabletop tasks (e.g., Levinson & Wilkins, 2006).

Our aim is to investigate how environmental affordances, cultural transmission, and individual cognition jointly shape the conceptualization of urban space. Specifically, by simulating generational learning in contrasting VR cityscapes, we test how spatial language adapts to the distinct affordances of different urban layouts, whether stable, environment-specific conventions emerge over generations, and whether individual conceptualizations are carried over into new environments. Participants navigated one of two environments—a grid-like (“Manhattan”) or an irregular, landmark-rich (“Barcelona”) layout—and produced route descriptions and maps that were passed to subsequent participants across transmission chains.

*H1: Environmental Affordances*: Our first hypothesis predicts that the conceptualizations underlying linguistic route descriptions will differ contingent on the topology of the environment. Individuals navigating the more structured and regular “Manhattan” environment will more likely rely on horizontal features for their route descriptions. In contrast, individuals navigating the irregular “Barcelona” environment will more likely employ words referring to properties of vertical landmarks when giving directions to another individual. We further predict that environment-aligned strategies will facilitate navigation performance, leading to faster task completion times.

*H2: Cultural Transmission*: According to our second hypothesis, differences in conceptualizations will be transmitted between individuals. Cultural transmission implies that individuals are influenced by the conceptualizations expressed in the route descriptions they receive, which shape their subsequent route descriptions. We additionally predict that these patterns may show evidence of cumulative change across generations (e.g. increase over generations of relevant descriptors given the environment), which would be consistent with cultural *evolution* beyond just transmission.

*H3: Individual Consistency and Carryover*: Our third hypothesis predicts that received conceptualizations create enduring cognitive models that shape subsequent spatial descriptions and actions. Consistency implies that individuals will express the same conceptualizations no matter the forms of expression (e.g. linguistic directions, or maps). Carryover implies that the conceptualizations will be coherently expressed not only within the same environment but also after an environmental switch within the same experiment. Such patterns would indicate relatively stable underlying representations formed during the task, rather than purely task-specific strategies.

Together, these hypotheses allow us to examine how environmental affordances, cultural transmission, and individual cognition interact to shape spatial conceptualization in urban environments.

## MATERIALS AND METHODS

This study was pre-registered, with details and any deviations described in Pre-Registration and Deviations section.

### Participants

Seventy-two participants (41 female, 31 male), mean age = 22.99 (*SD* = 2.85), participated in the study. The majority were students recruited from the participant pool of the Cognition and Behavior Lab at Aarhus University, Denmark. To avoid confounds related to major differences in language proficiency, only native speakers of Danish were included. All participants provided informed consent in accordance with local research ethics regulations, and received DKK 100 (∼€14) for their participation.

### Stimuli and Setup

The experiment was conducted using stereoscopic HTC Vive virtual reality equipment, enabling immersive interaction with complex environments while maintaining experimental control and reproducibility (Pan & Hamilton, 2018; Peeters, 2019).

Using the Unity 3D engine (version 2018.3.12f1), we constructed two virtual cityscapes, one for each of the two conditions: The “Manhattan” condition consisted of a regular, grid-like structure of broad, straight and perpendicular streets, while the buildings had low color-contrast and non-salient textures (see Figure 2, top row). In contrast, the “Barcelona” condition had the same number and relative positions of buildings, street intersections, and squares, but the horizontal layout was transformed to create irregular, winding streets, and the buildings had more saturated colors and salient textures (see Figure 2, bottom row).

**Figure 2.**
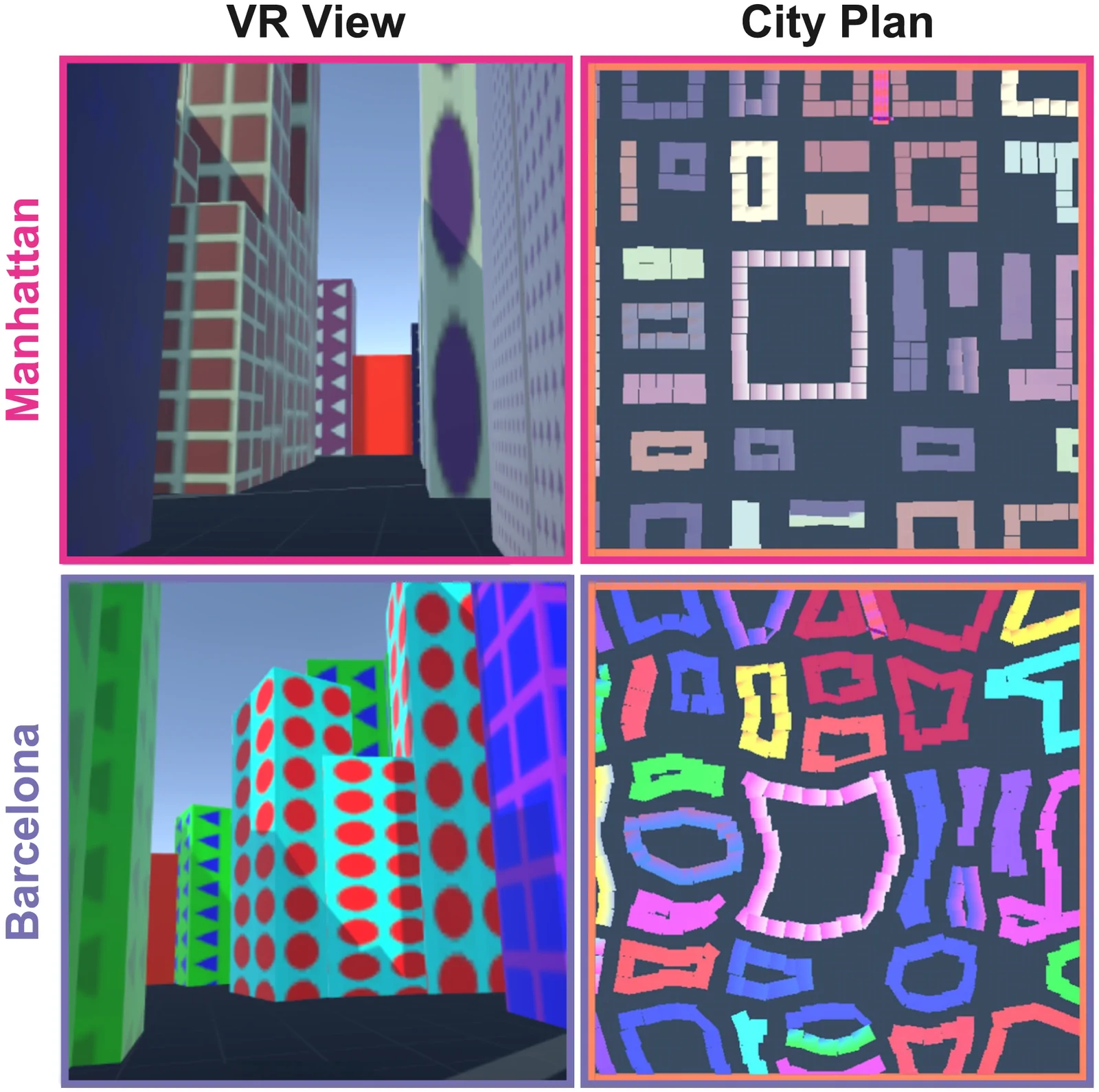
The two virtual city environments used in the experiment. The top row shows the “Manhattan” cityscape, characterized by a regular, grid-like street structure and low-salience buildings (left: representative first-person view; right: plan view). The bottom row depicts the “Barcelona” cityscape, featuring an irregular, winding street layout and high-salience buildings (left: representative first-person view; right: plan view). The environments were designed to differ in the relative salience of horizontal (street-based) and vertical (landmark-based) cues.

These manipulations were designed to create differences in the salience of horizontal (street-based) versus vertical (landmark-based) cues for navigation. Importantly, the contrast between environments was relative rather than absolute, allowing multiple strategies to be used in both conditions while differing in their degree of affordance.

### Procedure

Upon entering the lab, participants were randomly assigned to a condition, a transmission chain, and a generation. They then received instructions. Participants were escorted to the middle of an empty lab space, where they put on the virtual reality headset. Inside the virtual environments, they moved forward by pressing buttons on a handheld controller and steered the direction by turning either their head or their entire body. They received only visual feedback of the urban environment. Before the main experiment, participants completed a short training session to familiarize them with the controls, environment, and task. The training phase used the same environment as the subsequent experimental phase. In this session, they practiced navigating through the environment and locating a sample target flower. There were no time constraints on any of the tasks. All trials were initiated at the same position in the cityscape marked by a gate and all trials ended with the participant returning to this position.

After the training trial, participants were instructed to carry out three phases (see Figure 3). In Phase 1, participants read and memorized route directions provided by the previous participant in the chain. They then entered the VR environment to follow the directions to the target. The phase ended once the target was located and the participant returned to the starting point. In Phase 2, participants located a new target in the same environment without guidance, returned to the starting point, and provided written route directions for the next participant. In addition, they drew a map of the route on a digital tablet. In Phase 3, participants switched to the other environment (i.e., from “Manhattan” to “Barcelona” or vice versa), located a new target without directions, and again produced route descriptions and maps.

**Figure 3.**
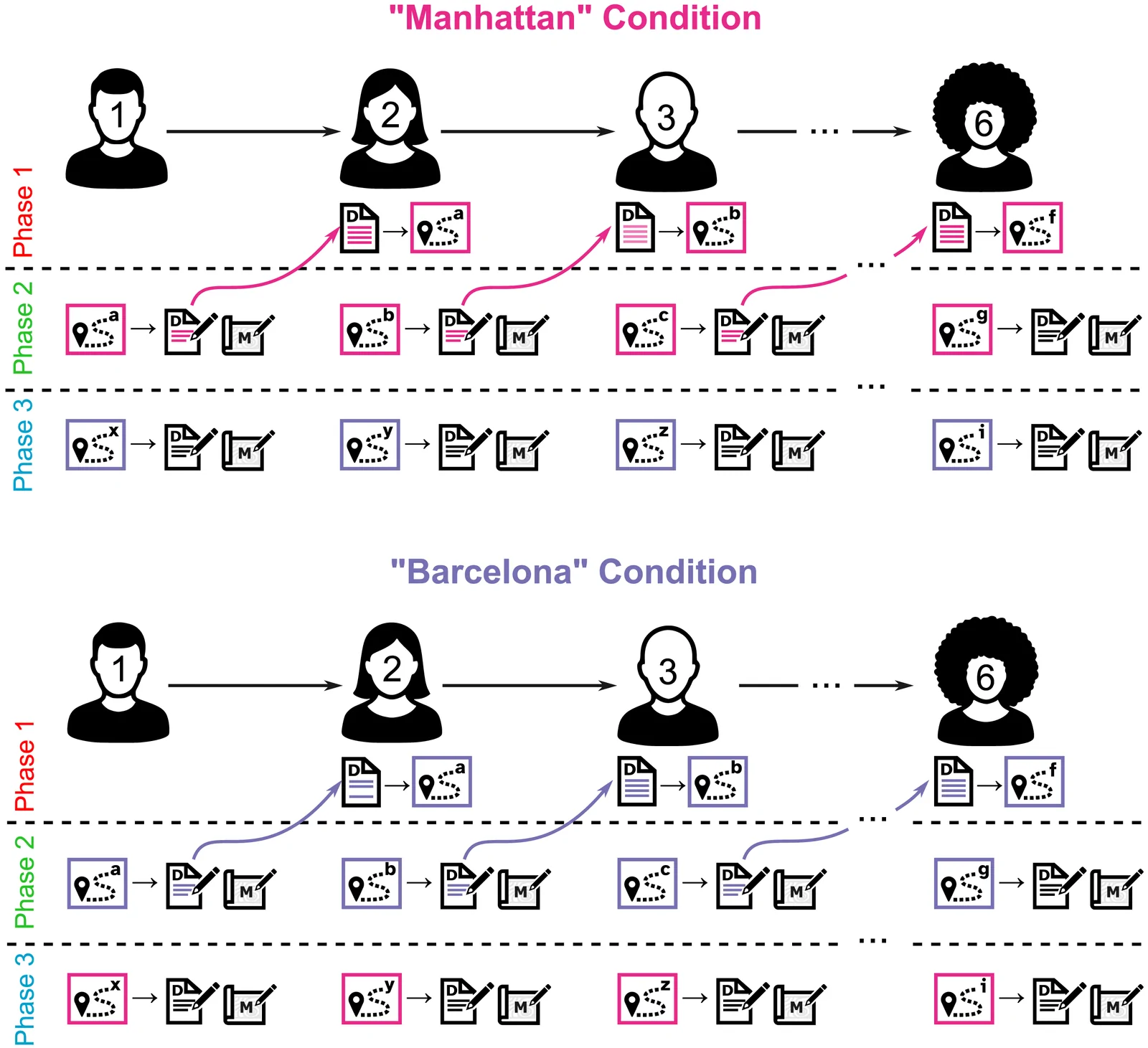
Experimental design. The transmission always starts in one of two environments and proceeds across generational chains (1 → 6). The top panel illustrates the Manhattan condition (pink), and the bottom panel illustrates the Barcelona condition (purple). Within each panel, rows represent phases and columns represent participants. Arrows indicate information flow across generations. Icons indicate the actions performed in each phase: pins represent target search in the current environment (pink/purple), superscripts (a, b, … / x, y, z) denote target locations, and other symbols represent written descriptions (D) and map drawings (M).

The task was self-paced, and participants were allowed to explore the environment as needed to produce an accurate description of the route. Thus, each participant completed one direction-follower task in Phase 1 and two direction-giver tasks in Phase 2 and 3, except first-generation participants, who completed only the direction-giving tasks. Participants typed their route directions using a laptop-based text editor, which was also used to present directions to subsequent participants. The maps were drawn on an Apple iPad using Evernote. Maps were not transmitted to the next participant, but collected to assess non-linguistic aspects of spatial representations across conditions and generations. The target flowers were placed at six different locations, counterbalanced across generations, chains and conditions. Target locations were systematically varied across generations such that each of the six possible locations appeared once in each phase of a chain. The position of the flower in participants’ Phase 1 was the same as in the previous participant’s Phase 2 so that the transmitted route description matched the target position (see superscripts in Figure 3). In all cases, the position required the same number of turns from the starting point. The experiment proceeded across six generations and six chains within each of the two conditions (“Manhattan” and “Barcelona”), with Phase 3 always involving a switch to the opposite environment to assess conceptual carryover.

### Analysis

#### Data Preprocessing.

To examine how participants’ conceptualizations varied across conditions and generations, we annotated their route descriptions, following the procedures adapted from Fay et al. (2018) and Fusaroli et al. (2012). First, we developed a coding scheme by identifying recurring concepts in the route descriptions: horizontal landmarks (e.g., streets, squares, intersections), vertical landmarks (buildings), numbers (e.g., first, second), colors (e.g., red, green), and textures (e.g., stars, circles). For example, horizontal descriptions typically included expressions such as “go straight and take the second left”, whereas vertical descriptions included references to landmarks, such as “walk towards the red building and turn left after it.” The full corpus of route descriptions and the coded dataset, including category annotations, are available in the OSF repository (https://osf.io/ve6kd). We then used an automated NLP procedure in R to identify and count the occurrence of words from each category in each route description. Counts were normalized by the total number of words in each description. The resulting dependent variables represent the relative frequency of each category within a given description. The same conceptual coding categories were applied to the map drawings. Three coders, two of which were naive to the hypotheses of the study, independently coded the presence or absence of the five categories (Fleiss’ *κ* = 0.86), as, unlike in linguistic descriptions where word tokens are discrete and countable, map drawings contain repeated geometric elements (e.g., lines, buildings) whose frequency is often ambiguous to interpret, making presence/absence coding more appropriate than frequency-based measures.

For analysis, the coded data were used in two ways: First, to compare conceptualizations across conditions, we collapsed the five categories into two broader groups: “Horizontal”, consisting of horizontal landmarks and numbers, reflecting attention to the horizontal structure of the environment, and “Vertical”, combining vertical landmarks, color, and texture, reflecting attention to vertical features of the environment (see Figure 4 for annotated examples from linguistic descriptions and map drawings). Second, we examined the individual categories to explore their contribution to these broader patterns. Detailed results for individual categories are reported in the Supplementary Material, with only key findings presented in the main text.

**Figure 4.**
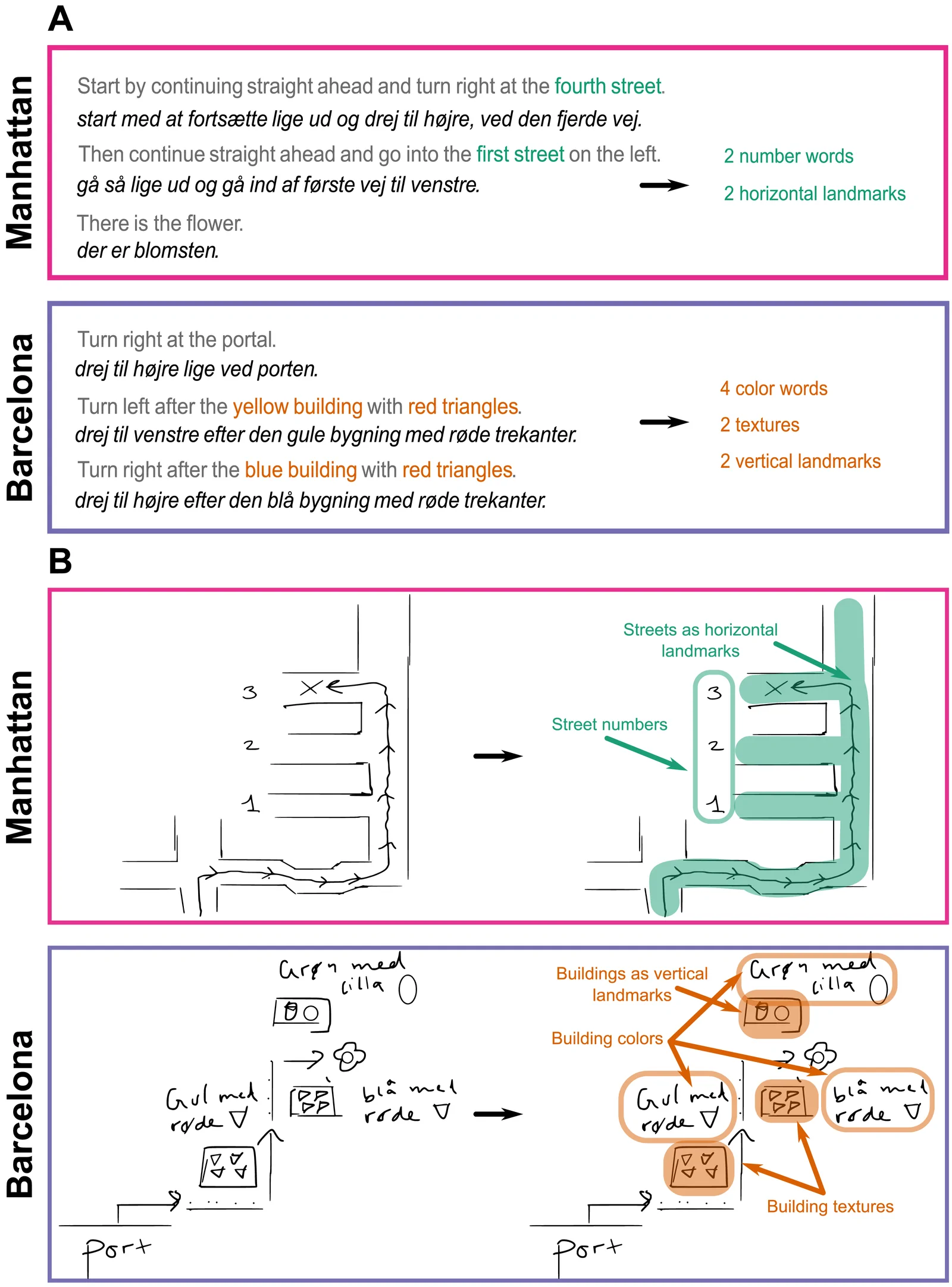
Examples of horizontal and vertical conceptual categories in linguistic descriptions and map drawings. (A) Example linguistic descriptions from the “Manhattan” and “Barcelona” conditions. Participants in the “Manhattan” condition primarily relied on horizontal references such as numbered routes and street sequences, whereas participants in the “Barcelona” condition relied more on vertical landmarks, colors, and textures. Underlined words indicate the coded descriptor categories, with English glosses shown above for clarity. (B) Example map drawings illustrating corresponding horizontal and vertical conceptualizations. Horizontal conceptualizations emphasized route numbers and street structure, whereas vertical conceptualizations emphasized buildings distinguished by colors, textures, and landmark features.

#### A Causal Diagram Approach.

To provide an overview of the analytical approach, we present in Figure 5 a causal diagram (Directed Acyclic Graph, or DAG, see Fusaroli et al., 2026; Pearl, 2009; and Roberts et al., 2020). In the diagram, nodes represent variables and arrows indicate hypothesized relationships between them. Panels A and B summarize analyses linking experimental variables (e.g., environment and transmitted information) to participants’ outputs. Panel C represents the flow of information across phases of the experiment.

**Figure 5.**
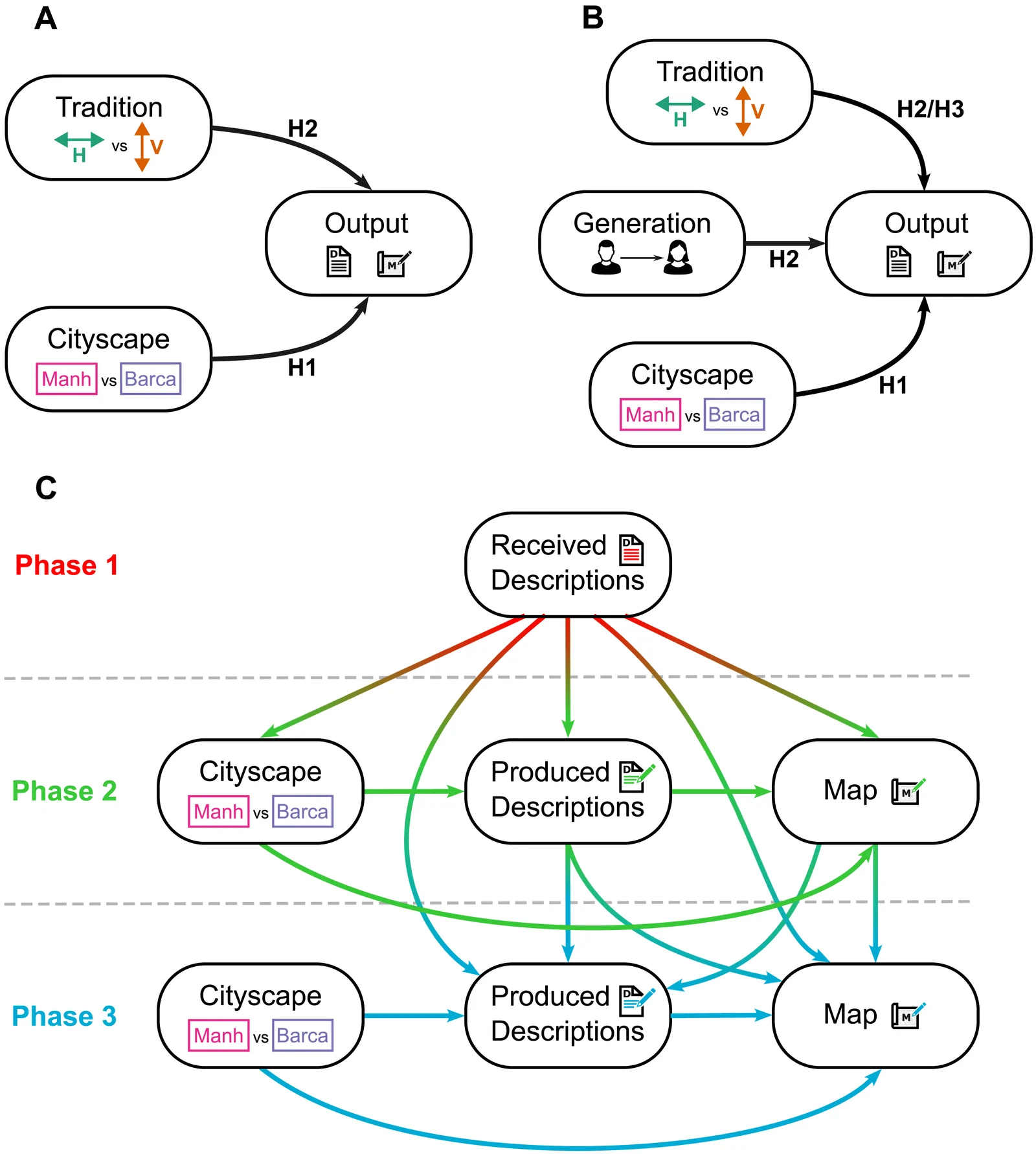
Causal diagrams (DAGs) for the main analyses. (A) Simplified diagram showing the influence of Cityscape (Manhattan vs. Barcelona) and Tradition (descriptor bias in received directions from previous participants) on participants’ outputs (relative use of horizontal vs. vertical descriptors). (B) Extended diagram including Generation, capturing changes across generations. (C) Diagram of information flow across phases, showing how received directions, environment, and participants’ own outputs relate across the experiment. Colored arrows indicate phase-specific pathways. H1, H2, and H3 correspond to the study’s three hypotheses.

#### Statistical Analyses of Phase 2: Environmental Affordances and Cultural Transmission.

To test for the main effect of Environmental Affordances (H1) and Cultural Transmission (H2), we fitted a Bayesian hierarchical model to the Phase 2 data, examining the effect of cityscape (Manhattan vs. Barcelona) and its interaction with generation. For linguistic data, the outcome variable was the relative frequency of descriptor categories (horizontal vs. vertical) within each route description. Because these proportions are bounded between 0 and 1 and include many zeros, we used a zero-inflated beta likelihood. This modeling approach is consistent with recent discussions emphasizing the value of generative models for improving inferential reliability in psychological science (Haines et al., 2025). Full model specifications, priors, and robustness checks are reported in the Supplementary Material and associated OSF repository.

Hypotheses were evaluated based on model predictions, with main effects assessed marginalizing across generations. For the map data, annotations captured the presence or absence of descriptor categories rather than their frequency. Accordingly, we used a comparable hierarchical model with a Bernoulli likelihood. We additionally analyzed individual descriptor categories using the same modeling approach (see Supplementary Material for details).

#### Statistical Analyses of Phase 3: Environmental Affordances vs. Cultural Transmission.

To assess the interplay between immediate Environmental Affordances (H1) and the carryover of previously acquired conceptualizations (H2/H3), we analyzed Phase 3 data, in which participants switched to a different environment, and compared these to Phase 2 data, where the received directions matched the environment. Here, we use the term “tradition” to refer to the route descriptions received from previous participants within a transmission chain, which serve as the cultural input influencing subsequent participants’ behavior.

We extended the model from Phase 2 to include the interaction between Tradition, Environment, Descriptor Type, and Generation. Map data were modeled using the same approach (see Supplementary Material for full model specification).

#### Statistical Analyses of Cultural Transmission and Individual Consistency.

To examine the mechanisms of Cultural Transmission (H2) and Individual Consistency (H3), we modeled the direct flow of information between participants, testing whether a participant’s outputs (language and maps) were predicted by the input they received and their own prior outputs. This approach allowed us to assess both cultural transmission between participants and consistency within individuals across modalities and environments. We fitted a series of Bayesian hierarchical models predicting participants’ outputs in Phases 2 and 3 as a function of received input and previously produced content.

For Phase 2 language, the outcome variable was the number of words belonging to each descriptor type (horizontal vs. vertical) relative to the total number of words, modeled using a binomial likelihood. For Phase 2 maps, we modeled the presence or absence of descriptor categories using a Bernoulli likelihood. These models tested both transmission from received directions and cross-modal consistency with participants’ own language production.

For Phase 3 language, we examined whether previously acquired conceptualizations persisted after the environmental switch. Predictors included received directions (Phase 1), participants’ own Phase 2 language, and their Phase 2 maps. For Phase 3 maps, we modeled feature presence using the same approach, including predictors from both prior language and map production.

Together, these models allow us to assess how conceptual patterns are transmitted between participants, maintained within individuals, and transferred across modalities and across environments.

#### Time to Solve Analysis.

Finally, to evaluate the functional consequences of these linguistic patterns, we analyzed task completion times to test whether environment-specific language (H1) was more communicatively efficient and whether this efficiency changed over generations (H2). We fitted a Bayesian hierarchical shifted lognormal model to participants’ task completion times when following received directions in Phase 1, a distribution appropriate for positively skewed reaction time data. The model included fixed effects for tradition and its interaction with generation, allowing us to estimate differences in task duration across environments and potential changes over generations. Random effects for chains were included to account for variability across transmission sequences. Full model specification and priors are reported in the Supplementary Material.

#### Pre-Registration and Deviations.

The study was initially pre-registered (https://aspredicted.org/xjkj-qzz7.pdf). The core components of the pre-registered design were preserved: the experimental conditions, the iterated learning paradigm, and sample size. Following pre-registration, refinements were made to the theoretical framing and statistical analysis. While the core hypotheses remained consistent, they were formalized into three explicit predictions (H1: Environmental Affordances, H2: Cultural Transmission, and H3: Individual Consistency). The pre-registered terminology (“holistic” vs. “figurative”) was refined into the more operational “horizontal” and “vertical” categories, based on a more detailed coding scheme.

The main deviation concerned the statistical analysis, where Bayesian hierarchical models were used instead of the initially planned models. This change was necessary because the pre-registered models did not adequately capture key properties of the data, including zero inflation.

While the pre-registration specified a categorical (binary) conceptualization variable, the final analyses represent conceptualization as the relative frequency of categories within each description. This extension was motivated by the observation that participants’ responses frequently contained multiple conceptual elements, making a binary classification insufficient to capture the full variation in the data.

In addition, we conducted two analyses not specified in the pre-registration: i) an analysis of information flow across participants to examine cultural transmission (H2) and individual consistency across modalities and contexts (H3); and ii) an analysis of task completion times (H1 and H2). Finally, due to the loss of essential documentation regarding the spatial cognition task and its scoring procedure, we were unable to reliably interpret individual differences in spatial cognition abilities and therefore could not include this analysis.

#### Statistical Software and Technical Implementations.

All models were implemented using a brms, cmdstanr, and Stan workflow in R (Bürkner, 2017; Carpenter et al., 2017; Gabry et al., 2025). Model quality was assessed using prior predictive checks, posterior predictive checks, and standard diagnostics to ensure adequate model fit, convergence (e.g., absence of divergences), and limited autocorrelation. We also verified that prior specifications did not unduly influence posterior estimates. Results are reported using Evidence Ratios (ER), defined as the posterior probability of the directed hypothesis relative to all alternatives (including the null hypothesis). As ER is a continuous measure without a fixed threshold, values above 3 are interpreted as indicating at least weak evidence. We also report posterior probabilities, defined as the probability that an effect is above (or below) zero. All plots displaying model results show raw data overlaid with posterior means, with error bars representing standard errors of the posterior distributions.

## RESULTS

### Environmental Affordances (H1, Phase 2)

A summary of the main results across hypotheses, modalities, and conditions is provided in Table 1.

**Table 1.** Summary of model results across hypotheses, modalities, and conditions.

**Hypothesis**	**Modality**	**Condition / Comparison**	**Estimate**	**95% CI**	**ER**
H1: Environmental Affordances	Language	Manhattan (Horizontal > Vertical)	3.7%	[−1.6%, 8.9%]	12.8
	Language	Barcelona (Vertical > Horizontal)	5.2%	[−4.0%, 11.7%]	12.4
	Maps	Manhattan (Horizontal > Vertical)	16.5%	[10.4%, 22.6%]	>1000
	Maps	Barcelona (Vertical > Horizontal)	9.3%	[3.4%, 15.1%]	614
H2: Cultural Transmission	Language	Barcelona (Decrease in Horizontal)	−4.3%	[−13.8%, 13.0%]	5.1
	Language	Manhattan (No clear trend)	—	—	∼1
	Maps	Both conditions (Flat trend)	—	—	0.99
H2 & H3: Tradition vs. Environment	Language	Manhattan (Tradition effect: Horizontal)	2.7%	[−2.2%, 7.7%]	6.4
	Language	Barcelona (Tradition effect: Horizontal)	−2.3%	[−32%, 7.7%]	1.24
	Maps	Manhattan (Tradition effect: Horizontal)	2.1%	[−30.7%, 73.4%]	4.2
	Maps	Barcelona (Tradition effect: Vertical)	28.3%	[−29.9%, 82.4%]	5.1
H2 & H3: Individual Transmission	Language	Barcelona → Manhattan (Horizontal carryover)	12.8%	[1.3%, 34.4%]	80.6
	Language	Manhattan → Barcelona (Vertical carryover)	12.5%	[−2.6%, 37.9%]	17.3
	Maps	Cross-environment consistency	86.7%	[65.0%, 97.9%]	>1000
H1: Communicative Efficiency	Task Duration	Manhattan (Horizontal vs. Vertical)	1.67 s	[−0.17, 4.33]	23.1
	Task Duration	Barcelona (Horizontal vs. Vertical)	0.51 s	[−1.81, 3.46]	1.81
H2: Communicative Efficiency	Task Duration	Manhattan (Generational slope)	−0.32 s	[−2.48, 1.57]	1.83
	Task Duration	Barcelona (Generational slope)	−0.18 s	[−3.89, 2.85]	1.11

*Linguistic Descriptions (Figure 6A):* We found a clear effect of the urban environment on the types of descriptors used in linguistic route descriptions, reflected in an interaction between cityscape and descriptor type. Participants in “Manhattan” used more horizontal than vertical descriptors, whereas participants in “Barcelona” used more vertical than horizontal descriptors.

**Figure 6.**
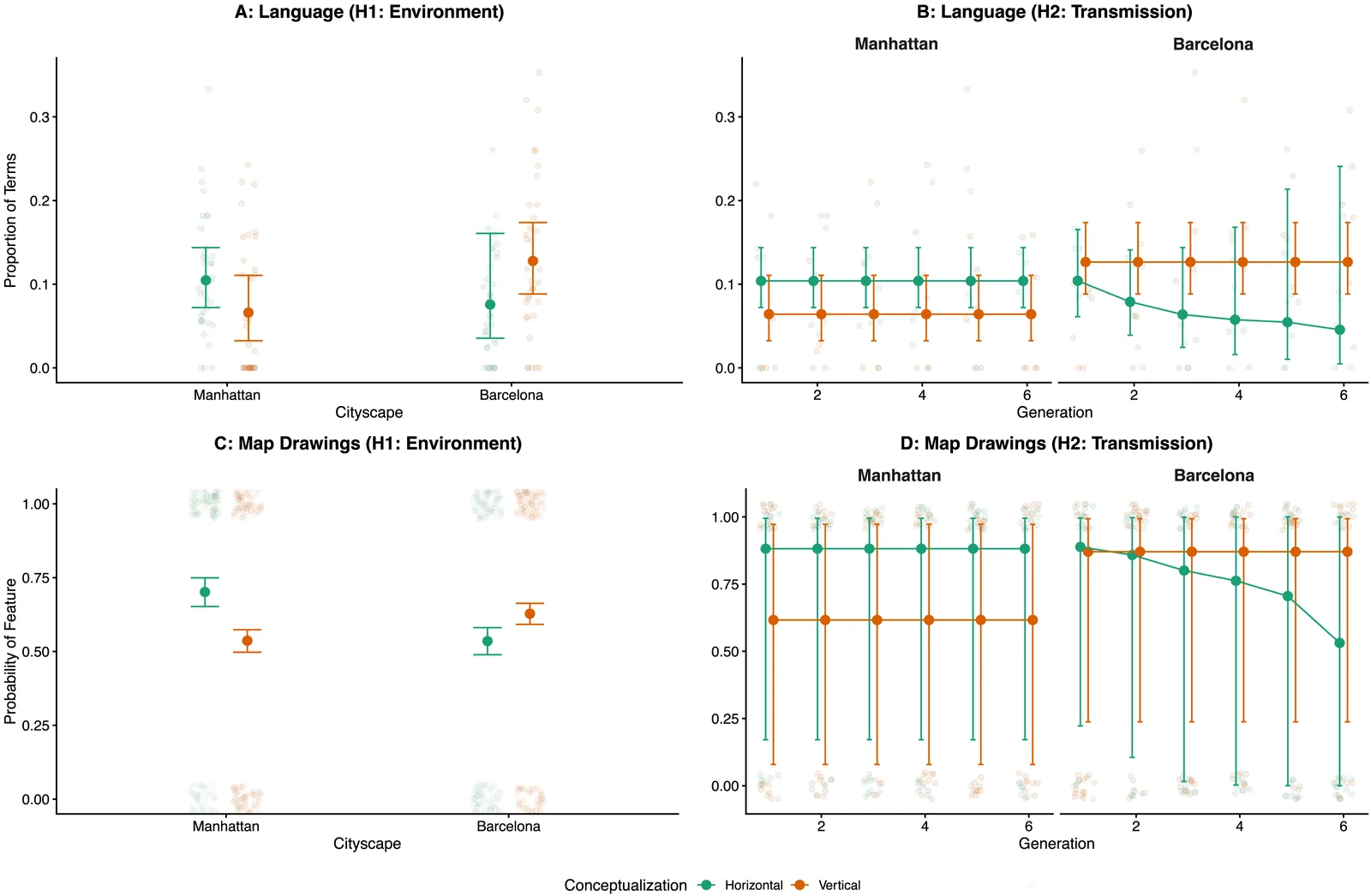
Effects of cityscape affordances and cultural transmission. (A) Linguistic descriptions: cityscape influences descriptor use. Participants in “Manhattan” rely more on horizontal descriptors, whereas participants in “Barcelona” rely more on vertical descriptors, consistent with H1. (B) Linguistic descriptions across generations: horizontal descriptors decrease in “Barcelona,” while remaining stable in “Manhattan.” Vertical descriptors remain relatively stable across conditions. (C) Map drawings show similar but less precise patterns. (D) Map drawings show no clear generational trends, with only weak and inconsistent changes across conditions.

*Map Drawings (Figure 6C):* Map drawings showed the same pattern, with stronger effects. Participants in “Manhattan” maps included more horizontal than vertical elements, whereas participants in “Barcelona” maps included more vertical than horizontal elements.

Analyses of individual descriptor categories showed that these effects were primarily driven by greater use of color in the “Barcelona” condition and greater use of number in the “Manhattan” condition (see Supplementary Material).

### Cultural Transmission (H2, Phase 2)

Having established that the environment shapes spatial language (H1), we next examined whether these patterns changed across generations, as would be expected under cumulative cultural processes. Overall, we found limited evidence for systematic increases in descriptor use across generations, and only weak evidence for condition-specific changes.

*Linguistic Descriptions (Figure 6B):* We found limited evidence for differential generational trends across conditions. Rather than showing increasing trends, the observed pattern was primarily driven by a decrease in horizontal descriptors in the “Barcelona” condition, while vertical descriptors remained relatively stable and no clear trends were observed in the “Manhattan” condition.

*Map Drawings (Figure 6D):* For the map drawings, we found no evidence of systematic change across generations. Generational trends were largely flat across both conditions, with no reliable differences across cityscapes or descriptor types.

Exploratory analyses of individual descriptor categories suggested more localized patterns with horizontal descriptors increasing in “Manhattan” and vertical descriptors increasing in “Barcelona” (see Supplementary Material).

### Cultural Transmission and Carryover Effects (H2 & H3, Phase 2 & 3)

While Cultural Transmission (H2, Phase 2) examined how the current environment shaped spatial descriptions, the following analysis examines the relative contribution of received tradition versus immediate environmental affordances, leveraging the environmental switch in Phase 3. We compared the relative impact of received tradition (carryover from prior input) and immediate environmental affordances (current cityscape).

*Linguistic Descriptions (Figure 7A, B):* We found evidence for effects of received tradition, particularly within the “Manhattan” cityscape. Within the “Manhattan” environment, a “Manhattan” tradition led to greater use of horizontal descriptors compared to a “Barcelona” tradition, whereas this effect was weak or absent in the “Barcelona” environment.

**Figure 7.**
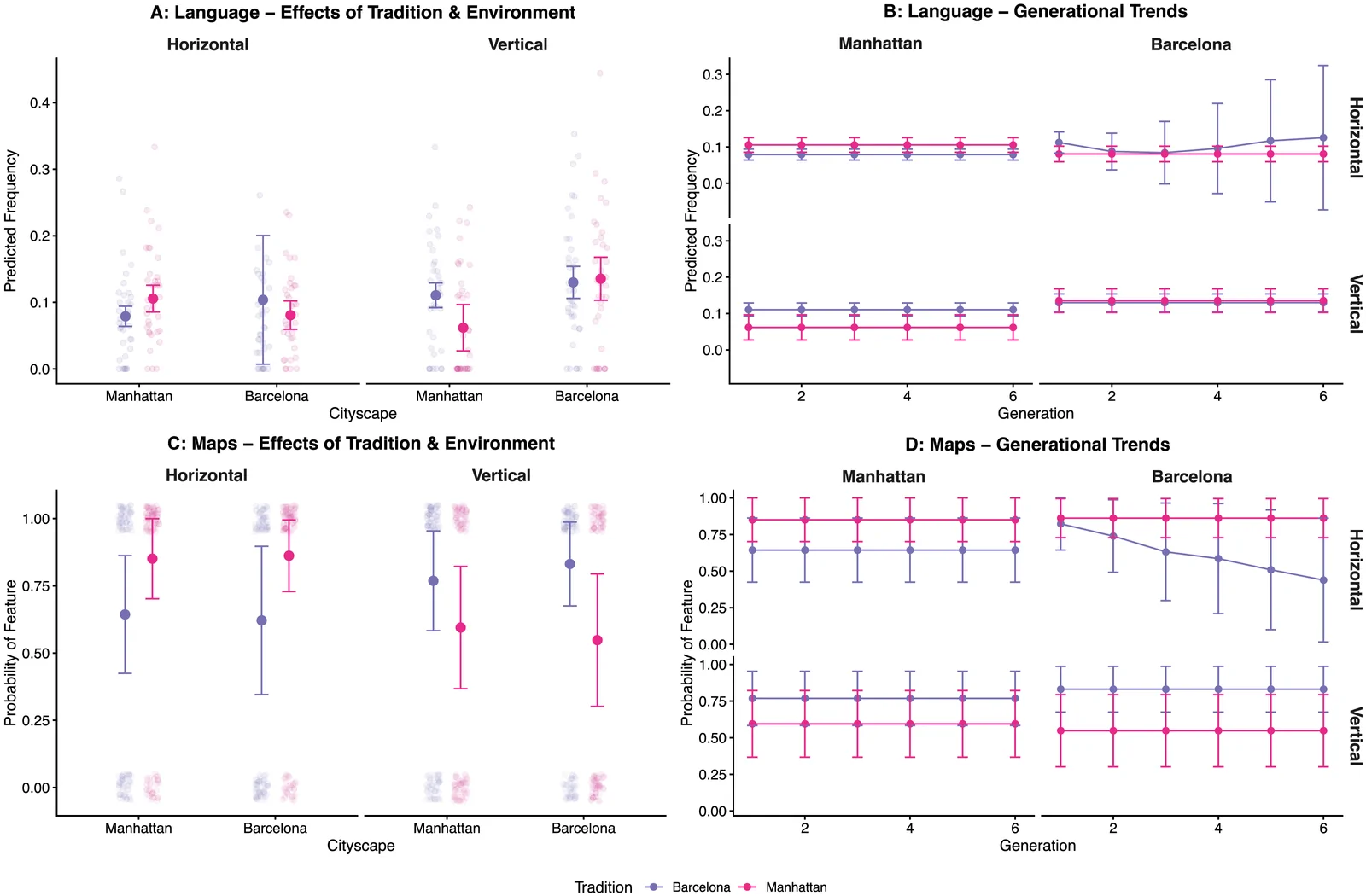
Effects of tradition, environment, and generational dynamics (H2 & H3). (A) Linguistic descriptions: both received tradition and environment influence descriptor use. “Manhattan” traditions promote horizontal descriptors, while “Barcelona” traditions promote vertical descriptors, particularly within the “Manhattan” environment. (B & D) Generational trends in language and maps. Across both modalities, descriptor frequencies remain largely stable across generations, indicating transmission without strong cumulative change. (C) Map drawings show similar but less precise patterns.

We also found effects of the current environment on language. Navigating “Manhattan” increased the use of horizontal descriptors, whereas navigating “Barcelona” increased the use of vertical descriptors, although these effects varied depending on the received tradition.

*Map Drawings (Figure 7C, D):* Map drawings showed similar patterns to the linguistic results, but with greater uncertainty. Tradition effects were directionally consistent across conditions, with “Manhattan” traditions tending to promote horizontal features and “Barcelona” traditions promoting vertical features. However, these effects were more variable and less reliable across conditions.

Effects of the current environment on maps were weaker and not reliable, and no robust differences emerged across environments or descriptor types.

Importantly, these effects did not increase systematically across generations, indicating that both received tradition and the immediate environment influenced behavior, but these influences remained stable rather than cumulative across transmission chains.

Exploratory analyses of subcategories showed that these tradition effects were most apparent in the “Manhattan” cityscape, where participants inheriting a “Barcelona” tradition used more color and vertical landmarks, and fewer numeric cues (see Supplementary Material).

### Cultural Transmission and Individual Consistency: Mechanistic Analyses (H2 & H3)

While evidence for cumulative change across generations was limited, mechanistic analyses revealed strong evidence for direct cultural transmission between individuals (H2). These analyses also allowed us to test intra-individual consistency across modalities and carryover across environments (H3).

*Cultural Transmission at the Individual Level (Figure 8)*. Across conditions, higher use of descriptors in received directions (Phase 1) reliably predicted higher use of the same descriptors in participants’ produced directions. This effect was evident both within the same environment (Phase 2) and after switching to a new environment (Phase 3). In Phase 3 language, received directions increased the use of horizontal descriptors in the “Barcelona–Manhattan” sequence and vertical descriptors in the “Manhattan–Barcelona” sequence, indicating strong cross-environment carryover. Similarly, in Phase 2 maps, received directions strongly influenced map content across environments.

**Figure 8.**
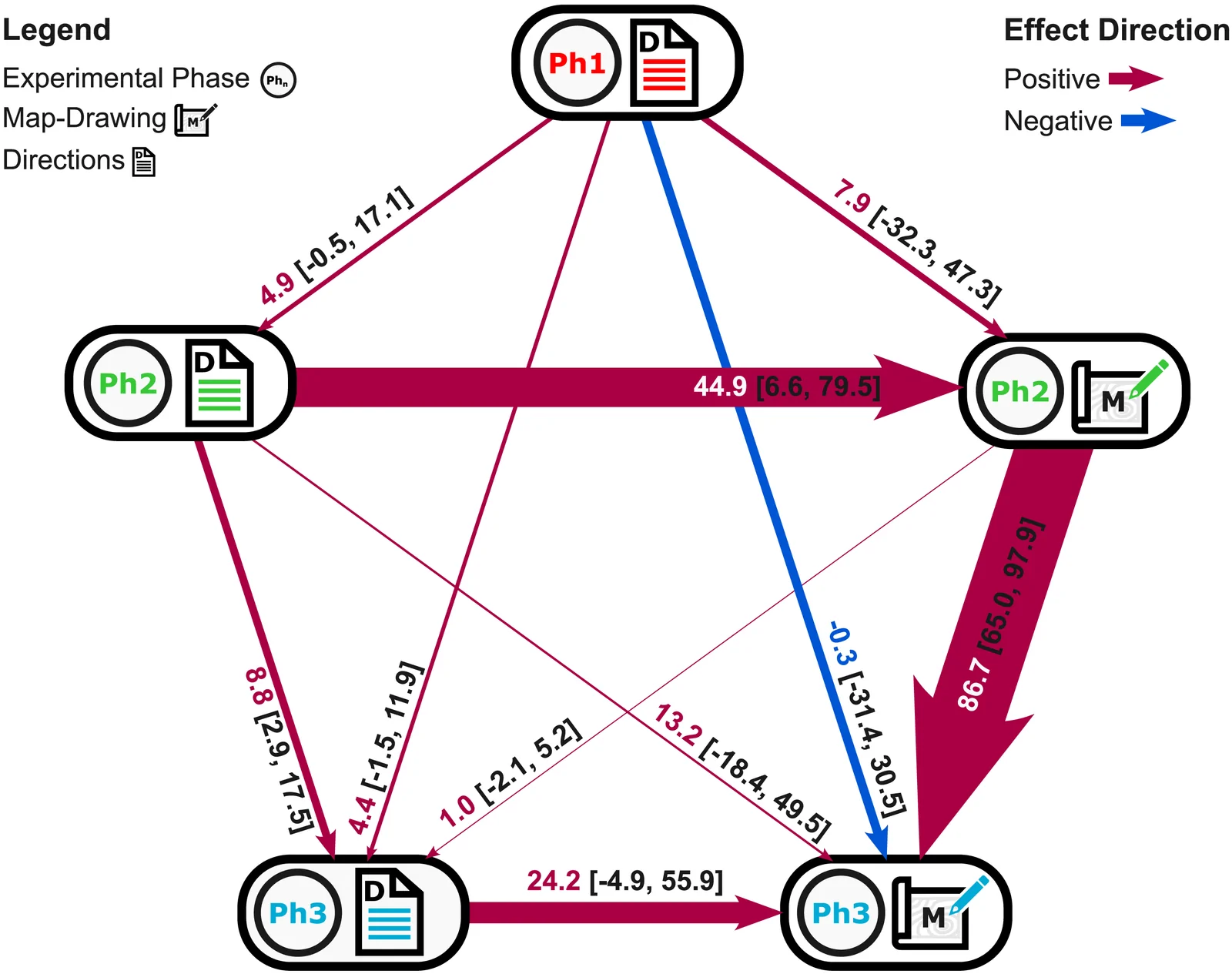
Directed Acyclic Graph (DAG) of information flow. This graph integrates the causal model of information flow between participants’ direction-giving and map-drawing behaviors, along with conditional estimates across experimental conditions. The arrows represent changes in the predicted relative frequency of descriptor categories, indicating how changes in one participant’s output affect subsequent behaviors in the transmission chain. Edge thickness reflects effect magnitude (absolute change in relative frequency), while color indicates direction (maroon = positive, blue = negative). Numbers on the edges indicate mean estimates with 95% credible intervals in square brackets. The strongest effects are observed from Phase 2 directions to Phase 2 maps and from Phase 2 maps to Phase 3 maps, indicating stable transmission within and across phases.

*Internal Coherence (Figure 9)*. Participants showed systematic consistency both across modalities and across environments. Within the same task, map coding reflected information present in participants’ own directions (Phase 2 across both cityscapes, Phase 3 particularly within the “Manhattan” tradition). Across environments, linguistic descriptions in Phase 3 reflected conceptual biases from Phase 2, even after switching environments. Map coding showed particularly strong cross-environment coherence.

**Figure 9.**
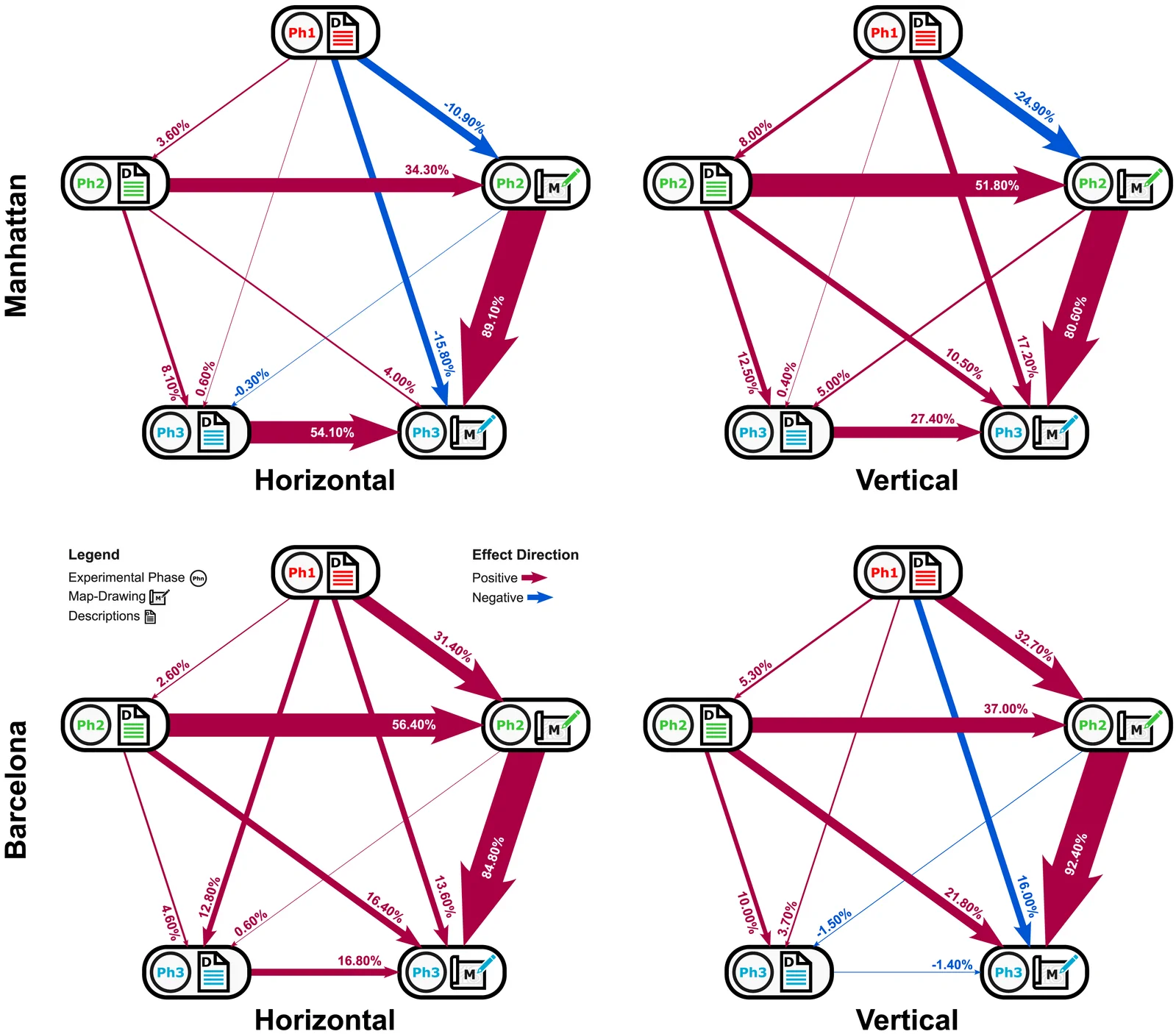
Conditional Directed Acyclic Graphs (DAGs) of information flow across environments and descriptor types. These graphs reproduce the causal model of information flow between participants’ direction-giving and map-drawing phases, with estimates stratified by environment (rows) and descriptor type (columns). The arrows represent changes in predicted relative frequency of descriptor categories, indicating how changes in one participant’s output affect subsequent behaviors in the transmission chain. Edge thickness reflects effect magnitude (absolute change in relative frequency), while color indicates direction (maroon = positive, blue = negative). Numbers on the edges indicate mean estimates (percentage change in predicted relative frequency). The four panels correspond to the two environments (“Barcelona”, “Manhattan”) crossed with the two descriptor types (horizontal, vertical), illustrating how transmission strength varies across conditions.

*Generational Enhancement of Carryover*. We also examined whether these effects increased across generations. Evidence for such increases was weak. In “Barcelona” traditions, some descriptors showed small trends toward increased alignment over generations, but these effects were small and uncertain. Map coding showed similarly weak patterns. No systematic generational trends were observed in the “Manhattan” condition.

Together, these analyses show that conceptual patterns are strongly transmitted between individuals and remain consistent within individuals across modalities and environments. However, these effects do not systematically amplify across generations, remaining stable rather than cumulative.

### Communicative Efficiency and Environmental Fit (H1 & H2)

To examine whether communicative efficiency changed over generations (H2) and whether it depended on the fit between descriptor type and environmental affordances (H1), we analyzed participants’ task completion times (Figure 10). We found no evidence that task duration decreased across generations, with effects remaining near zero across both environments. However, linguistic content was related to task efficiency, particularly in the “Manhattan” condition. In “Manhattan,” a higher frequency of horizontal descriptors was associated with faster task completion, whereas a higher frequency of vertical descriptors was associated with slower completion. Direct comparisons indicated that horizontal descriptors were more efficient than vertical descriptors in “Manhattan”. In “Barcelona,” the evidence was inconclusive, with no reliable difference in efficiency between descriptor types.

**Figure 10.**
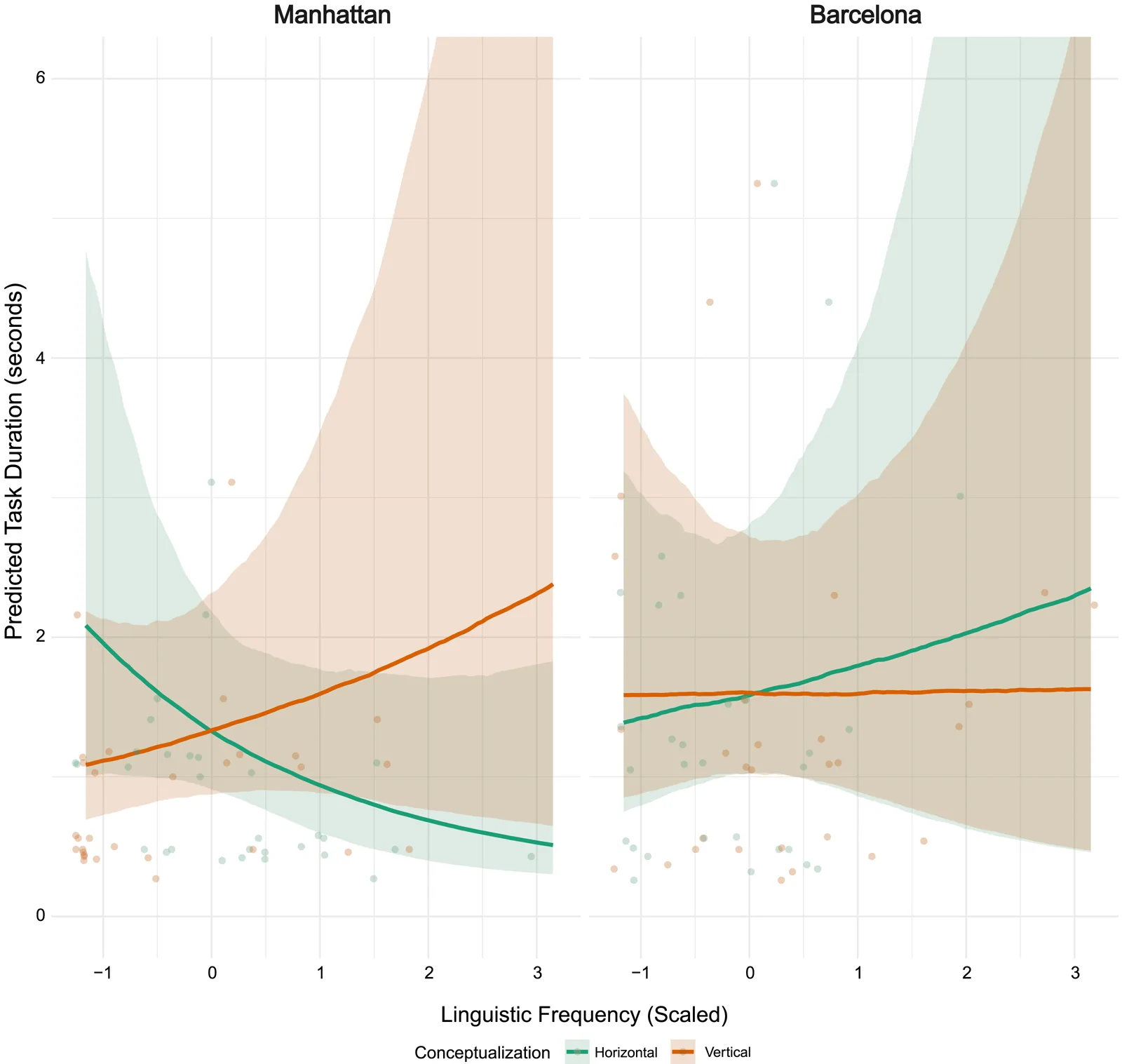
Effect of linguistic frequency on task duration. Predicted task completion times (seconds) as a function of the frequency of horizontal (green) and vertical (orange) descriptors in each cityscape. Shaded areas represent 95% credible intervals. In “Manhattan” higher use of horizontal descriptors is associated with faster task completion, whereas in “Barcelona” no clear difference is observed between descriptor types.

These results suggest that, in some contexts, environment-aligned language may facilitate more efficient communication, although we did not observe improvements in efficiency across generations.

## DISCUSSION

The present study examined how spatial conceptualizations emerge, stabilize, and are transmitted in urban environments by experimentally disentangling the contributions of environmental affordances (H1), cultural transmission (H2), and individual cognitive consistency and carryover (H3). Using a virtual reality (VR) paradigm combined with a transmission design, we simulated generational learning in two contrasting cityscapes—a grid-like environment (“Manhattan”) and an irregular, landmark-rich environment (“Barcelona”). Participants produced route descriptions and sketch maps across generations, allowing us to examine how conceptual strategies were shaped by environmental structure, linguistic input, and individual carryover. The discussion is structured around these three components, before considering broader implications for embodied and cultural accounts of spatial language.

### Environmental Affordances Shape Conceptualization (H1)

The results provide strong support for H1: the physical layout of the urban environment shapes how people describe it. In the grid-like “Manhattan” environment, participants used more horizontal descriptors (e.g., street numbers, intersections) in both language and maps. Conversely, in the irregular, landmark-rich “Barcelona” environment, participants used more vertical descriptors (e.g., landmarks, colors, textures). These patterns persisted even when participants switched environments (Phase 3), where they rapidly adapted their descriptions and maps to match the new environmental structure. These effects were primarily driven by increased use of numerical descriptors in “Manhattan” and color-based descriptors in “Barcelona”. These findings suggest that spatial language functions as a flexible interface between cognition and environment. The use of immersive VR allowed participants to generate situated descriptions based on active navigation rather than abstract reasoning. In this sense, the study provides experimental evidence that environmental structure shapes spatial language use, consistent with one component of the linguistic niche hypothesis, namely that environmental structure can shape spatial language (Lupyan & Dale, 2010, 2016). However, we note that the linguistic niche hypothesis ultimately concerns long-term population-level adaptation, which our study, demonstrating short-term individual-level effects without cumulative accumulation, does not directly address.

Using environment-appropriate conceptualizations was associated with more efficient communication. In particular, in the “Manhattan” environment, horizontal descriptors were associated with faster task completion, whereas vertical descriptors were associated with slower performance. Conversely, mismatches between descriptor type and environment were associated with slower navigation. These findings suggest that alignment between language and environmental structure can facilitate coordination, consistent with prior work linking linguistic strategies to environmental constraints (e.g., Fusaroli et al., 2012, 2014a, 2014b; Fusaroli & Tylén, 2012; Nölle et al., 2018, 2020; Palmer et al., 2017).

### Cultural Transmission (H2)

The second hypothesis—that environmental effects would be transmitted and potentially amplified across generations—received support for cultural transmission (person-to-person information flow), but not for cumulative change. Variations in descriptor use in received directions (Phase 1) reliably predicted participants’ subsequent behavior, both within the same environment (Phase 2) and after switching environments (Phase 3), even when controlling for intermediate performance. These results indicate that conceptualizations were socially transmitted, above and beyond the influence of the physical environment. Participants did not merely respond to environmental affordances, but also reproduced and propagated previously encountered linguistic patterns. However, we did not observe a systematic increase in environment-specific descriptors across generations. Instead, we only observed a localized decrease in horizontal descriptors in the “Barcelona” condition, while patterns in “Manhattan” remained stable. This asymmetry suggests that the two environments may differ in how readily their affordances support stable conceptual alignment. The regular structure of “Manhattan” may support more immediate alignment, whereas the more complex “Barcelona” environment may require repeated exposure before consistent patterns emerge. Analyses of individual descriptor categories suggested localized trends consistent with our predictions, but these effects were generally weak.

Overall, the results indicate strong social learning and high-fidelity cultural transmission, but not cumulative cultural evolution in the sense of progressive amplification or optimization. Instead, transmission appeared to preserve environment-specific patterns rather than amplify them. This suggests that, over the timescale of the experiment, cultural processes maintained stability rather than producing a directional change. This pattern aligns with recent work suggesting that cultural transmission often results in stabilization rather than cumulative transformation, although such outcomes may depend on task structure and learning conditions. For example, Acerbi and Mesoudi (2015) distinguish between high-fidelity transmission, which can support cumulative change, and reconstructive transmission, which often leads to stable convergence. Mesoudi (2021) further notes that cumulative selection is not a universal feature of cultural systems, and Acerbi et al. (2021) demonstrate how stability can emerge without progressive refinement. These accounts are consistent with our findings, where conceptual strategies remained stable across transmission chains rather than showing sustained directional change. More broadly, the pattern we observe—high-fidelity transmission without cumulative amplification—is consistent with what Smaldino (2026) terms “transformative selection,” whereby learned information is non-randomly modified within individual minds to become more consonant with existing cognitive categories and constraints. In this framework, the cognitive consistency we observe at the individual level (H3) is not merely a parallel finding but a mechanistic explanation for the absence of cumulative change (H2): individuals’ stable spatial representations function as cognitive attractors (Buskell, 2017) that preserve environment-specific conventions rather than progressively refining them.

### Individual Consistency as a Mechanism of Stability (H3)

The third hypothesis, concerning the stability of individual conceptualizations, was supported. Participants showed consistent patterns across modalities: descriptors used in verbal directions predicted the features represented in maps, suggesting a shared underlying representation.

Participants also showed consistency across environments: descriptions produced in Phase 2 influenced descriptions in Phase 3, even when controlling for received input. This indicates that individual conceptualizations can persist across environments, providing continuity within transmission chains. Such persistence likely contributes to reliable transmission: while influenced by environment and received input, individuals tend to reproduce previously established conceptualizations, thereby maintaining these patterns across participants. At the same time, this persistence may limit change: established conceptual models may favor stability over innovation. When participants switched environments, previously acquired linguistic patterns continued to influence their descriptions, indicating carryover with limited transformation.

Across analyses, linguistic descriptions and map drawings showed parallel—but not identical—patterns. Both reflected environmental affordances, with horizontal relations emphasized in “Manhattan” and vertical landmarks in “Barcelona.” However, differences between modalities emerged: linguistic descriptions appeared more flexible and responsive to input and environmental changes, whereas maps appeared more stable and sometimes retained traces of prior conceptualizations, even after environmental switches. This relative stability may also reflect the affordances and conventions of the representational medium itself, as map drawings impose structural constraints on how spatial information can be expressed and updated, potentially limiting flexibility compared to linguistic descriptions. These patterns suggest that language may function as a socially responsive channel of adaptation, while maps reflect more stable, perceptually grounded representations. Together, these findings highlight how spatial conceptualizations are shaped by both communicative processes and perceptual experience, contributing in different ways to the stabilization and transmission of shared representations.

This pattern resonates with accounts of cognitive maps that emphasize how spatial representations integrate information across multiple sources and modalities (Epstein et al., 2017; Mou, 2025). The consistency between linguistic descriptions and map representations may reflect shared reliance on such multimodal spatial representations, linking communicative and perceptual processes within a common representational framework.

At the same time, the results point to specific mechanisms operating at the level of individual cognition. Individuals can act as ‘bottlenecks,’ meaning that transmitted information undergoes selection at the level of individual cognition: some variants are preferentially retained over others (variant selection), while others are transformed to become more consistent with existing mental models and physical constraints (Bartlett, 1932; Griffiths et al., 2008; Smaldino, 2026). These processes can stabilize or reshape what is passed on.

### Distinctive Cityscapes and the Role of Embodiment

The asymmetric patterns observed between the “Manhattan” and “Barcelona” environments highlight an important nuance in how affordances are perceived and taken up in spatial conceptualization. In both Phase 2 and 3, the grid-like “Manhattan” environment showed relatively stable alignment between environment, tradition, and production, with participants consistently using horizontal descriptors. In contrast, the landmark-rich “Barcelona” environment showed more variable patterns and weaker carryover effects, suggesting that its affordances may be less immediately aligned with participants’ strategies. This asymmetry was also reflected in the efficiency analysis: in “Manhattan,” horizontal descriptors were associated with faster task completion, whereas no clear efficiency advantage was observed in “Barcelona.”

Together, these findings suggest that more regular environments may support more consistent and readily adopted conceptual strategies. This interpretation is consistent with previous works proposing that regular, predictable environments can support more uniform strategies and reduce cognitive demands (Choi et al., 2014; de Jong, 2010; Kirschner et al., 2018).

These differences point to how urban morphology may influence the ease and stability of spatial conceptualization. Our findings suggest that the distinctive features of environments can shape how quickly and consistently conceptual strategies are adopted. The immersive and embodied nature of the VR paradigm may have played an important role: participants actively navigated the environments rather than passively observing them, making sensorimotor experience part of the conceptualization process (Barsalou, 2008; Foglia & Wilson, 2013; Wellsby & Pexman, 2014). Such embodied interaction may help explain why more complex environments like “Barcelona” showed slower or less consistent alignment, and suggests that these dynamics may be less apparent in non-immersive experimental settings.

### Urban Design as a Catalyst for Cognitive-Cultural Niches

Our findings provide experimental insights into how urban form may shape cognitive and communicative processes. From a cultural evolutionary perspective, urban environments can be understood as a form of niche construction (Odling-Smee et al., 2003; Smaldino, 2026), in which the built environment shapes the selection pressures acting on spatial concepts and communicative strategies, which in turn influence how people interact with and modify those environments. The contrast between more rapid alignment in “Manhattan” and more gradual, variable patterns in “Barcelona” offers a controlled illustration—and a simplified microcosm—of how different urban structures may give rise to distinct communicative dynamics.

The relative efficiency and consistency observed in the grid-like environment are broadly in line with the notion of legibility proposed by Kevin Lynch (1960), which emphasizes how certain urban structures may be more readily organized and represented in cognitive maps. Our results suggest that grid-like layouts may support not only visual legibility, but also more consistent cognitive and communicative strategies. In our experimental setting, participants converged more readily on shared descriptors in such environments. This may help explain the persistence of grid plans in many urban contexts (e.g., Kostof, 1991), although such interpretations remain speculative beyond the scope of the present study.

By contrast, the “Barcelona” condition resembles more irregular urban environments, where navigation relies more heavily on local features. In such settings, coordination may depend more on shared experience and cultural transmission. Repeated interaction with such environments may support the development of shared landmark-based strategies, where salient features serve as common reference points for communication (Winter et al., 2008). This perspective resonates with ideas proposed by Jane Jacobs (1961), who emphasized that complex urban environments rely on locally developed, experience-based knowledge rather than imposed structure. Extending this logic, our findings suggest that such “local knowledge” may be supported by processes of transmission and stabilization in communication. The slower and more variable patterns observed in “Barcelona” may reflect the greater cognitive demands of such environments, although further research would be needed to establish such relationships.

From a practical perspective, these findings suggest that urban planning may shape not only physical spaces, but also environments that can influence how people conceptualize and communicate about space. Grid-like layouts may encourage more abstract, metric, and relational strategies, whereas landmark-rich, irregular environments may support more concrete, feature-based, and qualitative forms of reasoning. Recognizing that spatial layouts can shape the concepts and linguistic strategies people use to coordinate may contribute to more human-centered design approaches, helping align the built environment with everyday navigation and collective sense-making. More broadly, these findings speak to ongoing urban design debates, where the advantages of highly structured plans are weighed against the more intuitive, experience-based qualities of organically developed environments.

### Broader Implications for Spatial Language and Cultural Evolution

Our findings have several implications for theories of spatial language and cultural evolution, particularly regarding the mechanisms through which environmental structure can shape spatial language. In terms of the taxonomy proposed by Smaldino (2026), we observed evidence for both variant selection (environment-aligned descriptors were associated with more efficient communication in Manhattan) and transformative selection—individuals’ cognitive representations acted as attractors that pulled transmitted descriptions toward environment-specific patterns. Such processes are often discussed in accounts of language–environment co-adaptation (e.g., the linguistic niche hypothesis; Lupyan & Dale, 2010, 2016). However, we emphasize that the present study captures short-term, individual-level adaptation and transmission dynamics, rather than long-term population-level change.

Our results also refine models of cultural evolution by distinguishing between transmission and cumulative change. We observed high-fidelity social learning (cultural transmission) without strong evidence for cumulative amplification across generations. This suggests that, in stable environments and well-defined coordination tasks, cultural transmission may primarily function to preserve and maintain conventions rather than progressively refine them (Bisin & Verdier, 2011; Silvey, 2016). Cumulative change may instead depend on additional conditions, such as larger populations, longer timescales, or shifting environmental pressures that introduce new adaptive challenges (Derex & Mesoudi, 2020).

More broadly, this study highlights the role of the individual as a key component of the cognitive-cultural niche (Alinam et al., 2023). Individual cognitive consistency appears to contribute to the stabilization of conventions, supporting their reproduction across interactions and their transfer across contexts. This provides a potential mechanism for how cultural patterns persist in the face of environmental variability and individual differences.

In line with work on the cognitive foundations of culture (e.g., Bartlett, 1932; Edwards & Middleton, 1987; Romney & Moore, 1998), these findings suggest that the capacity of individuals to form relatively stable, cross-modal representations plays a central role in sustaining cultural systems—even when, as observed here, short-term dynamics are characterized more by stability than by cumulative innovation.

### Limitations and Future Directions

Several limitations of the present study point to directions for future research. First, the generational chains were relatively short (six generations), and lacked real-time interaction, which has been suggested to play an important role in communicative optimization and cumulative cultural evolution (Garrod & Doherty, 1994; Kirby et al., 2015). This fully linear, non-interactive structure did not include corrective pressures associated with real-time dialogue, such as clarification requests and repair (Fay et al., 2018). Consequently, the observed stability of the linguistic system may reflect the absence of pressures for progressive optimization rather than an inherent preference for stability. While short chains were sufficient to detect transmission and stabilization, future work with longer chains could help determine whether cumulative change emerges over extended timescales or whether the system stabilizes into a persistent equilibrium.

Second, the participant sample was culturally and linguistically homogeneous (native Danish speakers). Future studies could compare speakers from languages with different spatial reference systems (e.g., geocentric vs. egocentric; Levinson, 2003) to examine whether entrenched linguistic habits modulate or interact with environmental affordances in shaping spatial conceptualization.

A third limitation concerns the map-drawing task, which provided a valuable non-linguistic complement to linguistic data but also introduced interpretive challenges. While maps captured environmental affordance effects (H1) clearly in some cases, the binary feature coding (presence/absence) and variability in sketch interpretation introduced uncertainty when modeling transmission and carryover (H2, H3). Future research could employ more quantitative or automated analyses of spatial representation (e.g., computational shape analysis, spatial clustering, pointing and distance estimation tasks) to better capture the interaction between linguistic and non-linguistic conceptualizations.

Finally, although VR offers high experimental control and immersion, it remains a simplified abstraction of real urban experience. Future field-based or mobile-app implementations in real urban environments could help validate and extend the ecological validity of these findings.

## CONCLUSION

This study suggests that the way we talk about and conceptualize urban space emerges from a dynamic, multi-level system. The physical environment provides affordances that can make certain spatial strategies more efficient. Cultural transmission, operating through social learning, serves as a conduit, passing these strategies from person to person. And the individual mind, with its tendency toward internal coherence and stability, contributes to this process by entrenching conceptualizations and carrying them across tasks and contexts. By integrating virtual reality with methods from cultural evolution, we provide controlled experimental evidence for the interplay between the city, the community, and the cognitive agent. Our findings suggest that urban landscapes can be understood as “cognitive-cultural niches,” where the built environment and social practices interact, with language serving both as a reflection of this relationship and as a mechanism through which it is maintained. As such, this study contributes to cognitive theory by highlighting the interactions between environmental structure, social influence, and individual cognition, and offers insights relevant to the design of future urban environments.

## ACKNOWLEDGMENTS

We would like to acknowledge our co-author Kristian Tylén, who sadly passed away before the final publication of this article. This work benefited greatly from his insight, generosity, and intellectual engagement, and we are deeply grateful for his contributions. We also thank Jesse Lynch for his help with the graphic design, Chris Haysen for his help with programming, and Amalie Lysgaard Andersen, Astrid Endrup Iversen, and Annamaya Halskov-Jensen for their help with data collection and Paul Smaldino for insightful discussions.

## FUNDING INFORMATION

The project was supported by seed funding from the Interacting Minds Centre (Aarhus University). KT was supported by the European Research Council Consolidator Grant project ‘eSYMb: The Evolution of Early Symbolic Behaviour’ (grant number: 101044626). JMN was supported by the Polish National Science Centre under grant agreement UMO-2023/49/B/HS2/02222.

## DATA AVAILABILITY STATEMENT

The full model implementation (including priors) and quality checks, together with the data used for the analyses, are accessible at Open Science Framework repository: https://osf.io/ve6kd.

## ETHICS AND CONSENT

The study received ethical approval from the Human Subjects Committee of the Cognition and Behavior Lab at Aarhus University, Denmark, and was conducted in accordance with local ethical guidelines and procedures.

## Supplementary Material


