# Supplementary material for: When the City Speaks: How Urban Form Shapes the Cultural Transmission of Spatial Conceptualization
Source: Open Mind (Camb). 2026 Aug 13;10:1310–37. doi: 10.1162/OPMI.a.380 (PMC13618751; doi:10.1162/OPMI.a.380)
Supplement: Supplementary file 1 [file opmi-10-1310-s001.pdf]

Supplementary Material for:

When the city speaks: How urban form shapes the cultural transmission of spatial conceptualization

Table of Contents

S1. Analysis of Phase 2: H1 (Environmental Affordances)

- S1.1. Deconstruction of model components
- S1.2. Descriptor sub-category Analysis: Environmental Affordances (H1)
- S1.3. Statistical Table: H1 - descriptor sub-categories

S2. Analysis of Phase 2: Cultural Transmission and Evolution (H2)

- S2.1. Sub-category Analysis: Cultural Transmission and Evolution (H2)
- S2.2. Statistical Table: H2 - Descriptor sub-categories

S3. Analysis of Phase 3: Individual Consistency and Carryover (H3)

- S3.1. Sub-category Analysis: Tradition and Carryover (H3)
- S3.2. Statistical Tables: H3 (Sub-categories)

S4. Supplementary Figures

S5. Mechanistic Analyses: Consolidated Statistical Tables (H2 & H3)

S6. Communicative Efficiency Analysis (H1 & H2)

- S6.1. Statistical Table: Generational Effects on Task Duration (H2)
- S6.2. Statistical Table: Linguistic Frequency Effects on Task Duration (H1)

N.b. Details on model implementation and model quality checks are available in the knitted markdown part available at:

https://osf.io/ve6kd

S1. Analysis of Phase 2: H1 (Environmental Affordances)

S1.1. Deconstruction of model components

A detailed examination of the model components reveals that the interaction between cityscape and descriptor type was primarily driven by the probability of using a descriptor category at all (i.e., the zero-inflation component). This effect was extremely strong (difference-of-differences: 42.1%, 95% CIs [11%, 76.5%], ER: 215, credibility: 0.99).In Manhattan, participants were substantially more likely to produce horizontal descriptors over vertical ones (mean difference = 36.6%, 95% CIs [14.3%, 58.3%]; ER: 1322). They were also more likely than participants in Barcelona to do so (10.5%, 95% CIs [-9.5%, 38%], ER: 4.3, credibility: 0.81). In Barcelona, participants did not reliably use more vertical than horizontal descriptors overall (5.5%, 95% CI [-16.3%, 32.9%], ER: 1.9, credibility: 0.65). However, they used vertical descriptors significantly more often than participants in Manhattan (31.6%, 95% CIs [8.6%, 54.6%], ER: 285, credibility: 0.996).By contrast, the conditional frequency of descriptors (given that they were used at all) showed weaker evidence for an interaction (4.8%, 95% CIs [-8%, 14.6%], ER: 5.1, credibility: 0.835). The clearest effect was in Barcelona, where, conditioned on being used, vertical descriptors appeared with higher frequency than horizontal ones (mean difference = 5.6%, 95% CIs [-5.7%, 13%], ER: 10.7, credibility: 0.92), and marginally more so than in Manhattan (2.6%, 95% CIs [-5.1, 9.5%], ER: 3.7, credibility: 0.79). In Manhattan, conditioned on being used, horizontal descriptors were not used more than vertical ones (-0.83%, 95% CIs [-8.2%, 5.8%], ER: 0.69, credibility: 0.41) and only marginally more than in Barcelona (0.0218%, 95% CIs [-8.7, 8.8%], ER: 3.4, credibility: 0.77).

S1.2. Descriptor sub-category Analysis: Environmental Affordances (H1)

To examine which specific categories of descriptors were driving the effects observed for the overarching horizontal versus vertical distinction, we re-ran the analyses on individual descriptor types: vertical descriptors (color, texture, and vertical landmarks) and horizontal descriptors (number and horizontal landmarks) as a function of cityscape and generation.

Vertical Descriptors. We found reliable evidence that color was used more in Barcelona than in Manhattan in both linguistic descriptions (ER = 4.67) and map drawings (ER = 5.3). However, texture and vertical landmarks did not show reliable differences across environments in Phase 2.

Horizontal Descriptors. We found reliable evidence that numbers were used more in Manhattan than in Barcelona in both language (ER = 6.14) and maps (ER = 4.21). Horizontal landmarks showed no reliable effect.

These findings indicate that the broader H1 results were largely driven by the contrast between color (Barcelona) and number (Manhattan).

S1.3. Statistical Table: H1 - descriptor sub-categories

Table S1.3 for H1. Bold are in reliably in accordance with our hypothesis

	Estimate Lang	ER Lang	Estimate Map	ER Lang
Color	**3.6% [-3.4%, 15%]**	**4.67**	**20% [-9.4%, 61.1%]**	**5.3**
Texture	1.7% [-2%, 10.2%]	2.65	13.6% [-24.4%, 63.5%]	2.83
Vertical Landmarks	1.4% [-3.7%, 7.2%]	2.59	16.1% [-45.1%, 67.8%]	2.47
Number	**2.1% [-5%, 7%]**	**6.14**	**25.6% [-38.9%, 77%]**	**4.21**
Horizontal Landmarks	0% [-5.1%, 9.4%]	1.44	12.3% [-44.5%, 61%]	2.13

S2. Analysis of Phase 2: Cultural Transmission and Evolution (H2)

S2.1. Sub-category Analysis: Cultural Transmission and Evolution (H2)

We tested whether environmental effects on individual descriptor categories were amplified across generations.

Vertical Descriptors. The hypothesis was supported for most vertical descriptors in language, with the generational trend for using color (ER = 4.52), texture (ER = 3.23), and vertical landmarks (ER = 6.52) being stronger in Barcelona. For maps, this was only true for vertical landmarks (ER = 3.27).

Horizontal Descriptors. The generational trend for using numbers (Language ER = 3.11; Map ER = 4.26) and horizontal landmarks (Language ER = 3.8; Map ER = 4.98) was reliably stronger in Manhattan than in Barcelona, both in directions and map drawings.

S2.2. Statistical Table: H2 - Descriptor sub-categories

Table S2.2 for H2. Estimates reliably in accordance with our hypothesis are in bold

	Estimate Lang	ER Lang	Estimate Map	ER Lang
Color	**6.3% [-3.6%, 29.5%]**	**4.52**	23.1% [-40.3%, 94.9%]	1.58
Texture	**3.6% [-2.1%, 20.2%]**	**3.23**	-8.3% [-73.4%, 80.8%]	0.41
Vertical Landmarks	**3.7% [-2.2%, 13%]**	**6.52**	**29.8% [-60.6%, 90.5%]**	**3.27**
Number	**0.7% [-6%, 13%]**	**3.11**	**22% [-67%, 81.6%]**	**4.26**
Horizontal Landmarks	**2% [-9.1%, 15.5%]**	**3.8**	**35.6% [-51.4%, 86%]**	**4.98**

S3. Analysis of Phase 3: Individual Consistency and Carryover (H3)

S3.1. Sub-category Analysis: Tradition and Carryover (H3)

We examined whether prior exposure (tradition) or the current cityscape better explained subcategory use in Phase 3.

Cityscape Effects. The cityscape effect was clearest in Manhattan, where participants used fewer vertical descriptors (color, texture, landmarks) and more horizontal landmarks when navigating Manhattan compared to Barcelona. In contrast, participants from a Barcelona tradition did not reliably adjust descriptors to the new cityscape.

Tradition Effects. The tradition effect was most visible in Manhattan, where exposure to Barcelona input led participants to use more color, texture, and vertical landmarks, and fewer number descriptors, compared to those in a Manhattan tradition. In the Barcelona cityscape, a Manhattan tradition prompted somewhat more use of number (ER = 3.3) and horizontal landmarks (ER = 4.7), but these effects were weaker.

S3.2. Statistical Tables: H3 (Sub-categories)

Table S3.2a for H3: Effect of Cityscape. Horizontal descriptors should show positive effects in Barcelona, negative in Manhattan. Vertical descriptors should show negative effects in Barcelona, positive in Manhattan. Effects in bold reliably accord with hypothesis H3.

	Estimate Lang	ER Lang	Estimate Map	ER Lang
Color<br>Barcelona Trad<br>Manhattan Trad	 -0.2% [-11.7% 6%]<br>**-4.3% [-8.4% 1.2%]**	 1.3<br>**18.2**	 -12.6% [-55% 24.8%]<br>-3.7% [-26.6% 13.8%]	 2.1<br>2.3
Texture<br>Barcelona Tradition<br>Manhattan Tradition	 -0.8% [-9.6% 2.7%]<br>**-1.5% [-3.8% 1.1%]**	 1<br>**7.1**	 -10.6% [-60.1% 27.5%]<br>-5.5% [-43.8% 26.3%]	 2<br>1.2
Vertical Landmarks<br>Barcelona Tradition<br>Manhattan Tradition	 0.2% [-4.7% 3.6%]<br>**-2% [-5.6% 2.5%]**	 1.54<br>**5.4**	 10.2% [-40.8% 63%]<br>0.6% [-47.5% 49%]	 1.85<br>1.04
Number<br>Barcelona Tradition<br>Manhattan Tradition	 0.3% [-7% 4.1%]<br>1% [-3.3% 5.4%]	 1.96<br>2.44	 4.3% [-53.1% 58.3%]<br>17.7% [-43.4% 69.1%]	 1.34<br>2.69
Horizontal Landmarks<br>Barcelona Tradition<br>Manhattan Tradition	 -1% [-10% 3.5%]<br>**1.2% [-4.7% 3.6%]**	 0.76<br>**3.14**	 -3.8% [-55.1% 39.3%]<br>-12.9% [-59.8% 38%]	 1.23<br>0.41

Table S3.2b for H3: Effect of Tradition. Horizontal descriptors should show positive effects in Barcelona, negative in Manhattan. Vertical descriptors should show negative effects in Barcelona, positive in Manhattan. Effects in bold reliably accord with hypothesis H3.

	Estimate Lang	ER Lang	Estimate Map	ER Lang
Color<br>Barcelona Env<br>Manhattan Env	 0.7% [-10.9% 7.1%]<br>**3.4% [-1.8% 7.2%]**	 1.9<br>**11.9**	 -16.3% [-58.3% 18.8%]<br>**7.4% [-11.3% 38.9%]**	 2.9<br>**3.7**
Texture<br>Barcelona Env<br>Manhattan Env	 -0.02% [-8.9% 3.5%]<br>**1% [-1.5% 3%]**	 1.4<br>**3.9**	 -8.1% [-59.7% 36%]<br>3% [-29.5% 37.3%]	 1.6<br>1.5
Vertical Landmarks<br>Barcelona Env<br>Manhattan Env	 0.6% [-4.7% 4.6%]<br>**1.6% [-2.4% 4.5%]**	 2<br>**4.6**	 -16.7% [-0.69 % 45%]<br>**26.3% [-30% 74.2%]**	 2.6<br>**4.7**
Number<br>Barcelona Env<br>Manhattan Env	 **11% [-6.2% 5.9%]**<br>**-1.9% [-6.2% 1.7%]**	 **3.3**<br>**6.1**	 7.9% [-53.2% 65.2%]<br>**-21.4% [-73% 38%]**	 1.6<br>**3.3**

Horizontal<br>Landmarks<br>Barcelona Env<br>Manhattan Env	 <br>-1.3% [-10.4% 3.4%]<br>-1% [-4.7% 3.1%]	 <br>0.6<br>2.4	 <br>**25.2% [-33.1% 69.2%]**<br>-16.1% [-63.2% 35%]	 <br>**4.7**<br>2.7

S3.3. Statistical Tables: Generational Trends in Carryover Effects

Table S3.3a for H2: Change in Cityscape Effect Over Generations. Horizontal descriptors should show positive effects in Barcelona, negative in Manhattan. Vertical descriptors should show negative effects in Barcelona, positive in Manhattan. Effects in bold reliably accord with hypothesis H2.

	Estimate Lang	ER Lang	Estimate Map	ER Lang
Color<br>Barcelona Trad<br>Manhattan Trad	 **-6.3% [- 29.5% 3.6%]**<br>0% [0% 0%]	 **4.5**<br>NA	 -23.1% [-94.9% 40.3%]<br>0% [0% 0%]	 1.6<br>NA
Texture<br>Barcelona Tradition<br>Manhattan Tradition	 **-3.6% [-20.2% 2.1%]**<br>0% [0% 0%]	 **3.2**<br>NA	 8.3% [-80.8% 73.4%]<br>0% [0% 0%]	 2.44<br>NA
Vertical Landmarks<br>Barcelona Tradition<br>Manhattan Tradition	 **-3.7% [-13% 2.2%]**<br>0% [0% 0%]	 **6.5**<br>NA	 **-29.8% [-90.5% 60.6%]**<br>0% [0% 0%]	 **3.3**<br>NA
Number<br>Barcelona Tradition<br>Manhattan Tradition	 **0.7% [-13% 5.9%]**<br>0% [0% 0%]	 **3.1**<br>NA	 **22% [-67% 81.6%]**<br>0% [0% 0%]	 **4.3**<br>NA
Horizontal<br>Landmarks<br>Barcelona Tradition<br>Manhattan Tradition	 <br>**2% [-15.5% 9.1%]**<br>0% [0% 0%]	 <br>**3.8**<br>NA	 <br>**35.6% [-51.4% 86%]**<br>0% [0% 0%]	 <br>**5**<br>NA

Table S3.3b for H2c: Change in Tradition Effect Over Generations. Horizontal descriptors should show positive effects in Barcelona, negative in Manhattan. Vertical descriptors should show negative effects in Barcelona, positive in Manhattan. Effects in bold reliably accord with hypothesis H2.

	Estimate Lang	ER Lang	Estimate Map	ER Lang
Color<br>Barcelona Env<br>Manhattan Env	 **-6.3% [-29.5% 3.6%]**<br>0% [0% 0%]	 **4.5**<br>NA	 -23.1% [-94.9% 40.3%]<br>0% [0% 0%]	 1.6<br>NA
Texture<br>Barcelona Env	 **-3.6% [-20.2% 2.1%]**	 **3.2**	 8.3% [-80.8% 73.4%]	 2.44

Manhattan Env	0% [0% 0%]	NA	0% [0% 0%]	NA
Vertical Landmarks<br>Barcelona Env<br>Manhattan Env	 **-3.7% [-13% 2.2%]**<br>0% [0% 0%]	 **6.5**<br>NA	 **-29.8% [-90.5% 60.6%]**<br>0% [0% 0%]	 **3.2**<br>0
Number<br>Barcelona Env<br>Manhattan Env	 **0.7% [-13% 5.9%]**<br>0% [0% 0%]	 **3.1**<br>NA	 **22% [-67% 81.6%]**<br>0% [0% 0%]	 **4.26**<br>NA
Horizontal Landmarks<br>Barcelona Env<br>Manhattan Env	 **2% [-15.5% 9.1%]**<br>0% [0% 0%]	 **3.8**<br>NA	 **35.6% [-51.4% 86%]**<br>0% [0% 0%]	 **5**<br>NA

S4. Supplementary Figures

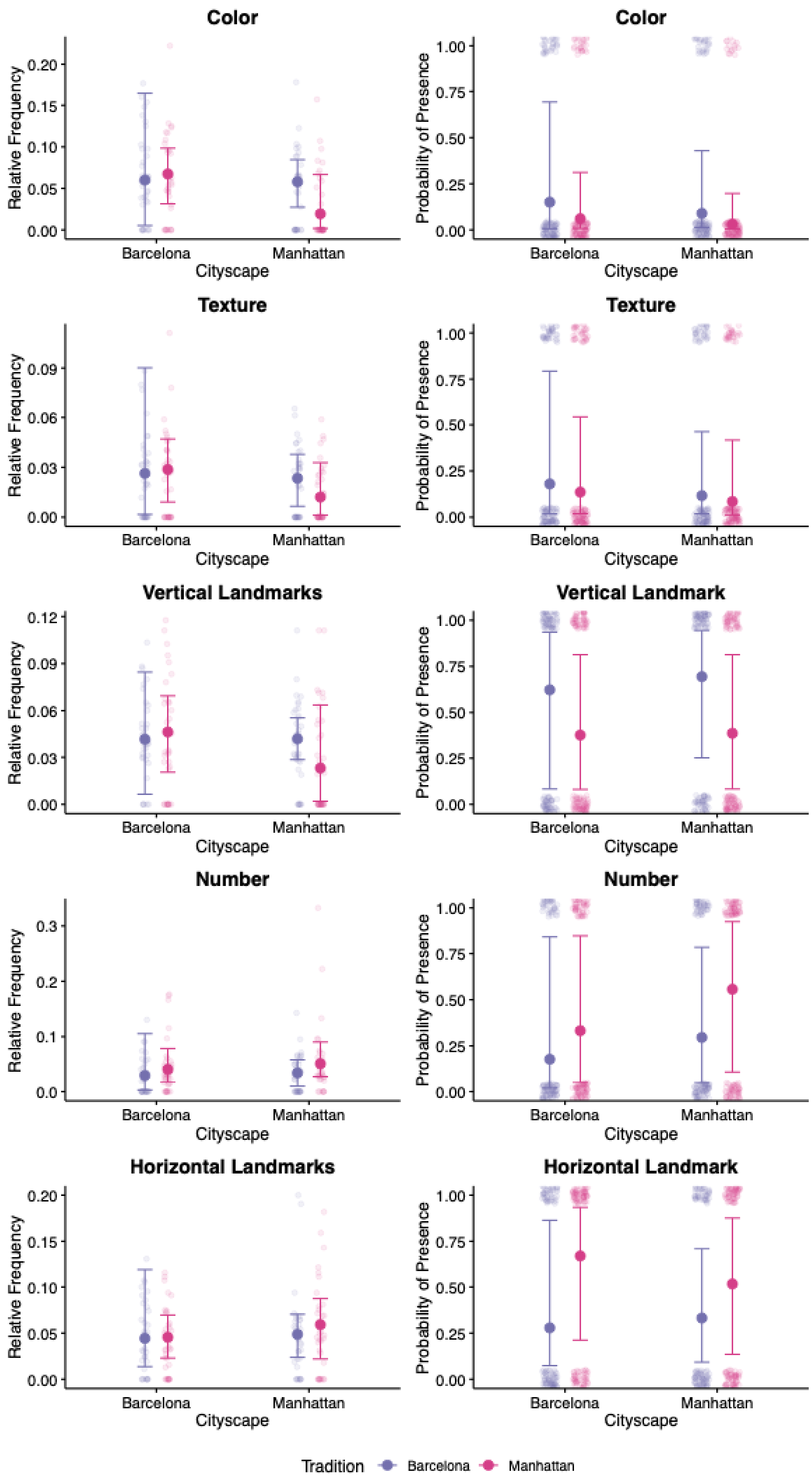

Supplementary Figure S4a. Effects of tradition and environmental affordances on descriptor subcategories (H3). This figure decomposes the aggregate horizontal and vertical conceptualizations (see Figure 7 in the main text) into specific descriptor categories: Color, Texture, and Vertical Landmarks (constituting the Vertical category), and Number and Horizontal Landmarks (constituting the Horizontal category). The *Left column* displays the predicted relative frequency of descriptors in linguistic route descriptions, while the *Right column* displays the predicted probability of feature presence in map drawings. The x-axis represents the current Cityscape (environmental affordances), while the colors represent the Tradition carried over from the previous environment (Purple = Barcelona tradition; Pink = Manhattan tradition). Points indicate posterior means with 95% credible intervals, overlaid on raw data points. The plots illustrate that the carryover effects observed in Hypothesis 3 are driven by specific features: the persistence of vertical conceptualizations in the Manhattan cityscape is largely driven by references to Color and Vertical Landmarks, whereas the persistence of horizontal conceptualizations is primarily driven by Number.

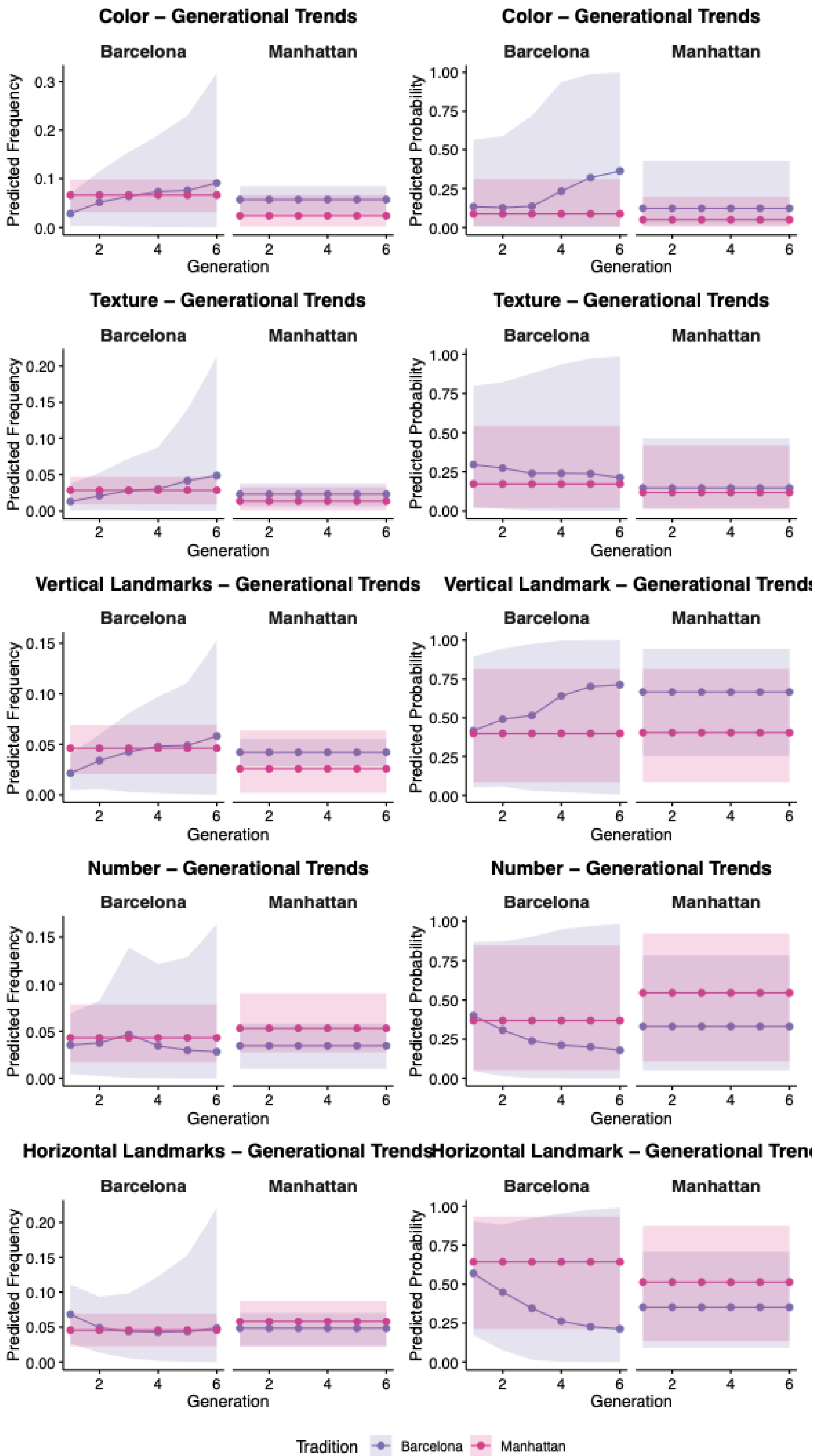
Color – Generational Trends
Texture – Generational Trends
Vertical Landmarks – Generational Trends
Vertical Landmark – Generational Trends
Number – Generational Trends
Horizontal Landmarks – Generational Trends
Horizontal Landmark – Generational Trend
Barcelona
Manhattan
Predicted Frequency
Predicted Probability
Generation
Tradition
Barcelona
Manhattan

Supplementary Figure S4b. Generational trends in descriptor subcategories across traditions and cityscapes (H2). This figure illustrates the evolution of spatial conceptualizations over six generations for specific descriptor categories. The *Left Column* displays the predicted relative frequency of descriptors in linguistic route descriptions (Zero-Inflated Beta model), while the *Right Column* displays the predicted probability of feature presence in map drawings (Bernoulli model). The plots are faceted by the current Cityscape (Barcelona vs. Manhattan) and colored by Tradition (Purple = Barcelona tradition; Pink = Manhattan tradition). Shaded areas represent 95% credible intervals around the posterior means. Consistent with the aggregate findings for Cultural Evolution (H2), the Barcelona cityscape (left panels) exhibits dynamic changes within the Barcelona tradition (purple lines): vertical features such as Color and Vertical Landmarks show an increasing trend over generations, while horizontal features such as Number and Horizontal Landmarks show a decreasing trend (dispreference). In contrast, the Manhattan cityscape (right panels) shows stable trajectories across generations, regardless of tradition, reflecting the asymmetric stability of the grid-like environment.

S5. Mechanistic Analyses: Consolidated Statistical Tables (H2 & H3)

Table S2. Consolidated differences in predicted probability for all mechanistic models corresponding to Figures 8 and 9 in the main text.

Outcome	Predictor	Condition	Estimate (%)	95% CI	Credibility	Cred	ER
P2 Language	P1 Language	Barcelona-Horizontal	2.6	"[-4.7	11.8]"	0.8	3.2
P2 Language	P1 Language	Barcelona-Vertical	5.3	"[-2.3	13.4]"	0.9	12.4
P2 Language	P1 Language	Manhattan-Horizontal	3.6	"[-5.4	14.1]"	0.8	4.1
P2 Language	P1 Language	Manhattan-Vertical	8.0	"[-3.5	55.6]"	0.8	5.1
P2 Language	P1 Language	Overall	4.9	"[-0.5	17.1]"	1.0	25.0
P2 Map	P1 Language	Barcelona-Horizontal	31.4	"[-46.9	92.6]"	0.8	4.6
P2 Map	P1 Language	Barcelona-Vertical	32.7	"[-44.5	95.5]"	0.8	4.8
P2 Map	P1 Language	Manhattan-Horizontal	-10.9	"[-83.9	62.2]"	0.3	0.5
P2 Map	P1 Language	Manhattan-Vertical	-24.9	"[-98.5	83.6]"	0.3	0.5
P2 Map	P1 Language	Overall	7.0	"[-32.3	47.3]"	0.6	1.7
P2 Map	P2 Language	Barcelona-Horizontal	56.4	"[-8.3	96.6]"	1.0	23.2

P2 Map	P2 Language	Barcelona-Vertical	37.0	"[-29.8	97.0]"	0.8	5.0
P2 Map	P2 Language	Manhattan-Horizontal	34.3	"[-22.2	94.3]"	0.9	8.2
P2 Map	P2 Language	Manhattan-Vertical	51.8	"[-62.4	99.6]"	0.9	6.6
P2 Map	P2 Language	Overall	44.9	"[6.6	79.5]"	1.0	74.5
P3 Language	P1 Language	Barcelona-Manhattan-Horizontal	12.8	"[1.3	34.4]"	1.0	80.6
P3 Language	P1 Language	Barcelona-Manhattan-Vertical	3.7	"[-3.4	13.8]"	0.9	5.8
P3 Language	P1 Language	Manhattan-Barcelona-Horizontal	0.6	"[-10.9	12.7]"	0.6	1.3
P3 Language	P1 Language	Manhattan-Barcelona-Vertical	0.4	"[-13.2	14.8]"	0.5	1.1
P3 Language	P1 Language	Overall	4.4	"[-1.5	11.9]"	0.9	12.1
P3 Language	P2 Language	Barcelona-Manhattan-Horizontal	4.6	"[-3.2	19.2]"	0.8	5.5
P3 Language	P2 Language	Barcelona-Manhattan-Vertical	10.0	"[0.7	24.9]"	1.0	54.6
P3 Language	P2 Language	Manhattan-Barcelona-Horizontal	8.1	"[1.0	19.9]"	1.0	63.5
P3 Language	P2 Language	Manhattan-Barcelona-Vertical	12.5	"[-2.6	37.9]"	0.9	17.3
P3 Language	P2 Language	Overall	8.8	"[2.9	17.5]"	1.0	362.6
P3 Language	P2 Map	Barcelona-Manhattan-Horizontal	0.6	"[-3.8	5.2]"	0.6	1.6
P3 Language	P2 Map	Barcelona-Manhattan-Vertical	-1.5	"[-8.3	4.8]"	0.3	0.4
P3 Language	P2 Map	Manhattan-Barcelona-Horizontal	-0.3	"[-4.1	3.3]"	0.4	0.8

P3 Language	P2 Map	Manhattan-Barcelona-Vertical	5.0	"[-3.3	20.4]"	0.9	6.5
P3 Language	P2 Map	Overall	1.0	"[-2.1	5.2]"	0.7	2.3
P3 Map	P1 Language	Barcelona-Manhattan-Horizontal	13.6	"[-64.6	93.5]"	0.7	2.1
P3 Map	P1 Language	Barcelona-Manhattan-Vertical	-16.0	"[-85.3	0.0]"	0.0	0.0
P3 Map	P1 Language	Manhattan-Barcelona-Horizontal	-15.8	"[-78.8	16.8]"	0.2	0.2
P3 Map	P1 Language	Manhattan-Barcelona-Vertical	17.2	"[-56.6	94.7]"	0.7	3.0
P3 Map	P1 Language	Overall	-0.3	"[-31.4	30.5]"	0.5	0.9
P3 Map	P2 Language	Barcelona-Manhattan-Horizontal	16.4	"[-48.4	94.1]"	0.6	1.8
P3 Map	P2 Language	Barcelona-Manhattan-Vertical	21.8	"[0.0	93.4]"	1.0	83.2
P3 Map	P2 Language	Manhattan-Barcelona-Horizontal	4.0	"[-71.8	79.9]"	0.6	1.3
P3 Map	P2 Language	Manhattan-Barcelona-Vertical	10.5	"[-70.4	96.2]"	0.6	1.3
P3 Map	P2 Language	Overall	13.2	"[-18.4	49.5]"	0.8	3.6
P3 Map	P3 Language	Barcelona-Manhattan-Horizontal	16.8	"[-64.2	96.9]"	0.6	1.6
P3 Map	P3 Language	Barcelona-Manhattan-Vertical	-1.4	"[-32.1	15.5]"	0.4	0.8
P3 Map	P3 Language	Manhattan-Barcelona-Horizontal	54.1	"[-0.3	99.6]"	1.0	32.6
P3 Map	P3 Language	Manhattan-Barcelona-Vertical	27.4	"[-11.9	92.7]"	0.9	11.5

P3 Map	P3 Language	Overall	24.2	"[-4.9	55.9]"	0.9	18.4
P3 Map	P2 Map	Barcelona-Manhattan-Horizontal	84.8	"[27.1	99.8]"	1.0	Inf
P3 Map	P2 Map	Barcelona-Manhattan-Vertical	92.4	"[48.9	99.9]"	1.0	Inf
P3 Map	P2 Map	Manhattan-Barcelona-Horizontal	89.1	"[48.7	99.7]"	1.0	7999.0
P3 Map	P2 Map	Manhattan-Barcelona-Vertical	80.6	"[14.2	99.7]"	1.0	180.8
P3 Map	P2 Map	Overall	86.7	"[65.0	97.9]"	1.0	Inf

S6. Communicative Efficiency Analysis (H1 & H2)

S6.1. Statistical Table: Generational Effects on Task Duration (H2)

Table S6.1 for H2. The table reports estimates for the change of time to solve over generation by cityscape, and their difference by cityscape. We expect time to solve to decrease over generations as participants evolve more effective descriptions.

Hypothesis	Mean	95% CIs	Cred	ER
H1: Barcelona slope over generation < 0	-0.182	[-3.89, 2.85]	0.53	1.1
H2: Manhattan slope over generation < 0	-0.322	[-2.48, 1.57]	0.65	1.8
H3: Difference (Bcn vs Man)	0.140	[-3.96, 4.04]	0.54	1.2

S6.2. Statistical Table: Linguistic Frequency Effects on Task Duration (H1)

Table S6.2 for H1 and H2. The table reports estimates for the relation between horizontal and vertical descriptions and time to solve by cityscape (estimates and measures of evidence). We expect horizontal descriptions to be positively related to time to solve in "Barcelona" and negatively in "Manhattan", and the opposite to be true for vertical descriptions.

Hypothesis	Mean	95% CIs	Cred	ER
H1a: Horizontal frequency slope in Barcelona > 0	0.51	[-1.52, 3.01]	0.69	2.26
**H1b: Horizontal frequency slope in Manhattan < 0**	**-1.11**	**[-3.06, 0.30]**	**0.95**	**17.02**
H1c: Difference (Bcn > Man)	1.62	[-0.94, 4.83]	0.90	8.57
**H2a: Vertical frequency slope in Manhattan > 0**	**0.56**	**[-0.54, 2.19]**	**0.82**	**4.45**
H2b: Vertical frequency slope in Barcelona < 0	-0.00	[-1.78, 1.45]	0.48	0.93
H2c: Difference (Man > Bcn)	0.57	[-1.35, 2.91]	0.71	2.39
H3: Bcn (Vertical < Horizontal)	0.58	[-1.81, 3.46]	0.64	1.81
H4: Man (Horizontal < Vertical)	1.67	[-0.17, 4.33]	0.96	23.10
